# Role of microglia autophagy and mitophagy in age-related neurodegenerative diseases

**DOI:** 10.3389/fnagi.2022.1100133

**Published:** 2023-04-26

**Authors:** Mingkai Lin, Hongwen Yu, Qiuyan Xie, Zhiyun Xu, Pei Shang

**Affiliations:** ^1^Department of Stomatology, Nanfang Hospital, Southern Medical University, Guangzhou, China; ^2^Nanfang Hospital, Southern Medical University, Guangzhou, China; ^3^Department of Neurology, Nanfang Hospital, Southern Medical University, Guangzhou, China; ^4^Breast Center, Department of General Surgery, Nanfang Hospital, Southern Medical University, Guangzhou, China

**Keywords:** autophagy, microglia, nanomedicines, neurodegenerative diseases (NDDs), neuroinflammation

## Abstract

Microglia, characterized by responding to damage, regulating the secretion of soluble inflammatory mediators, and engulfing specific segments in the central nervous system (CNS), function as key immune cells in the CNS. Emerging evidence suggests that microglia coordinate the inflammatory responses in CNS system and play a pivotal role in the pathogenesis of age-related neurodegenerative diseases (NDDs). Remarkably, microglia autophagy participates in the regulation of subcellular substances, which includes the degradation of misfolded proteins and other harmful constituents produced by neurons. Therefore, microglia autophagy regulates neuronal homeostasis maintenance and process of neuroinflammation. In this review, we aimed at highlighting the pivotal role of microglia autophagy in the pathogenesis of age-related NDDs. Besides the mechanistic process and the co-interaction between microglia autophagy and different kinds of NDDs, we also emphasized potential therapeutic agents and approaches that could be utilized at the onset and progression of these diseases through modulating microglia autophagy, including promising nanomedicines. Our review provides a valuable reference for subsequent studies focusing on treatments of neurodegenerative disorders. The exploration of microglia autophagy and the development of nanomedicines greatly enhances current understanding of NDDs.

## 1. Introduction

Neurodegenerative diseases (NDDs) are significantly intertwined with the disorders of multicellular interactions, cellular structure, and cellular function in the central nervous system (CNS) (Katsnelson et al., 2016). NDDs such as Parkinson’s disease (PD) have a tremendous impact on patients and their caregivers, adding to the personal and social economic burden (Martinez-Martin et al., 2012). In recent years, the growing incidence of NDDs arouses the awareness of the public and promotes the progression of research. For instance, Alzheimer’s disease (AD) is proved to be exquisitely associated with dementia (Nelson et al., 2011), and the prevalence of AD is expected to rise globally (Brodaty et al., 2011).

Microglia, the resident macrophages that are sensitive to brain injury, are the central part of the innate immune system in the CNS (Plaza-Zabala et al., 2017). As it can respond to pathophysiological insults *via* altering its morphology and differentiation states (Perry et al., 2010), microglia can be utilized as a diagnostic marker of the onset or progression of multiple neurological diseases. Furthermore, emerging evidence suggests that autophagy plays an essential role in NDDs. Since the microglia autophagy under normal circumstance maintains the physiological function and cellular homeostasis in CNS, microglia autophagy deficiency can cause the accumulation of misfolded proteins and other toxic substances that are packaged in intracellular inclusion bodies, and further result in the onset or progression of neurological disorders (Plaza-Zabala et al., 2017). As the treatment of NDDs is in an urgent need of in-depth study, the interactions between microglia autophagy and NDDs are worth to be explored.

In this review, we initially described the definition and mechanisms of autophagy. We further discussed the relationship between impaired autophagy and NDDs. Particularly, we focus on AD, PD, Huntington’s disease (HD), and amyotrophic lateral sclerosis (ALS). Meantime, novel therapeutic strategies of NDDs focusing on microglia autophagy are briefly outlined according to recent researches, which include drug development, clinical trial, and exploration of the molecular mechanisms.

## 2. Microglia autophagy

Autophagy is a major pathway for the removal of damaged organelles from cells (Proikas-Cezanne and Ktistakis, 2020). Lysosomes are the main organelles responsible for digesting and recycling of all types of macromolecules, while unnecessary or damaged intracellular material can be imported into lysosomes through autophagy. As a checkpoint of cellular homeostasis, autophagy is involved in the basal turnover of long-lived macromolecules or organelles to regulate energy, transmit intracellular signals, and protect cells against malfunctioning or damaged organelles (Plaza-Zabala et al., 2017; Heckmann and Green, 2019). As a result of extracellular or intracellular stress induced by cellular starvation, growth factor deprivation, endoplasmic reticulum (ER) stress, and pathogen infection, autophagy is stimulated to maintain cellular homeostasis in the CNS. Conversely, defective autophagy plays an indispensable role in human pathological process, including neurodegeneration, cancer and infectious diseases (He and Klionsky, 2009). During energy crisis, autophagy can be non-selective with absorbing generic cytoplasmic materials, or selective with removing damaged organelles, such as mitochondria, the ER, Golgi membranes and protein aggregates (Duan et al., 2022). In addition to previously described functions, microglia autophagy regularly removes harmful substances produced by neuronal cells and also promotes the conversion of microglia from M1-like to M2-like phenotype, the former of which strongly correlated with amyloid pathology while the latter shows neuroprotective effect (Komatsu et al., 2006; Matarin et al., 2015).

Depending on substrate selectivity and pathways to lysosome, microglia autophagy can be classified as partner-mediated autophagy, microasphagy, and macroevolgophagy (Li et al., 2012; Tasset and Cuervo, 2016; Xilouri and Stefanis, 2016). Macroautophagy plays a significant role in brain aging by taking over the autophagic pathway. Despite being distinct in morphology, all three pathways lead to the delivery of cargo to the lysosome for degradation and recycling (Yang and Klionsky, 2010). In the context of the CNS, autophagy in adult organisms plays an important role not only in neuronal development but also in maintaining homeostasis (Andres-Alonso et al., 2021; Kuijpers et al., 2021).

In non-canonical autophagy processes, components of the autophagy machinery are deployed to fulfill functions which do not involve lysosomal delivery of cytosolic entities. In recent years, there has been increasing evidence suggesting the existence of autophagy-like pathways, consisting of shared autophagy machinery and specific components that serve specific cellular settings or locations (Codogno et al., 2012; Dupont et al., 2017). These distinctive functions of autophagy proteins have been referred to as non-canonical autophagy, even though technically they do not involve “self-eating” process or non-canonical functions, which modulate host-pathogen interactions, regulate neuronal signaling, and contribute to anticancer immunity. Importantly, there are two pathways of non-canonical autophagy that have been studied extensively: LC3 (microtubule-associated protein light chain 3)-associated phagocytosis (LAP) and LC3-associated endocytosis (LANDO). LAP features the family of microtubule-associated proteins 1A/1B light chain 3 to phagosome membranes (Sanjuan et al., 2007; Martinez et al., 2011, 2015). Comparatively, LANDO is a novel form of receptor-mediated endocytosis (RME) and receptor recycling. LANDO features conjugation of endosomal membranes with LC3/GABARAP-family proteins. For microenvironment, LANDO is essential for preventing exacerbated accumulation of beta-amyloid (Aβ) and minimizing beta-amyloid-induced neuroinflammation in AD models (Heckmann et al., 2019, 2020).

### 2.1. Initiation and inhibition of autophagy in microglia

**FIGURE 1:**
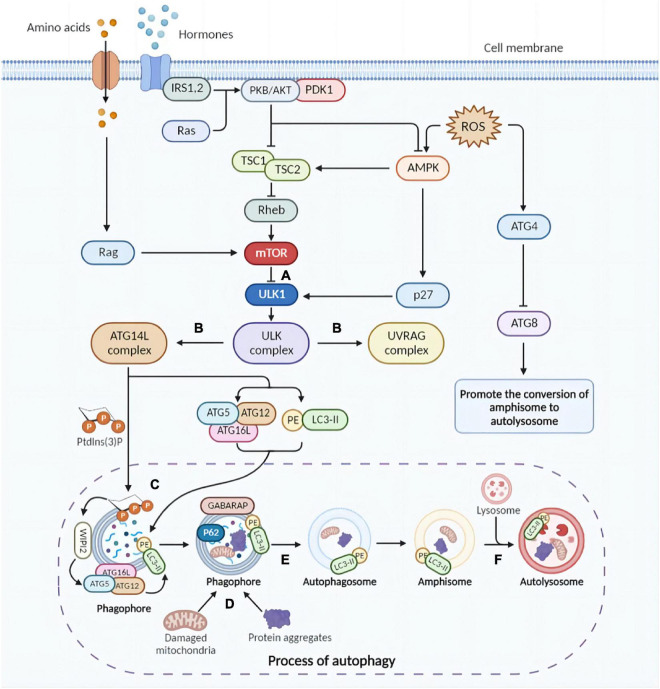
Brief molecular mechanisms of autophagy initiation and mTOR activation. **(A)** Physiologically, mTOR impedes autophagy by inhibiting the ULK complex (autophagy initiation complex). Under certain conditions, due to mTOR inhibition, the ULK complex is activated, triggering the initiation of autophagy. Furthermore, inflammatory factors activate AMPK, which promotes phosphorylation of ULK1 and facilitates the assembly of ULK complexes. **(B)** The ULK complex activates the UVRAG and ATG14L complexes. UVRAG complex is involved in the fusion of autophagosomes and lysosomes. ATG14L triggers the production of Ptdlns(3)P required for phagosome nucleation and expansion. Subsequently, ATG5-ATG12 conjugation and LC3 lipidation are activated. **(C)** The ATG5-ATG12-ATG16L complex localizes on the phagosomal surface with the assistance of PtdIns(3)P, activating ATG3 and lipidating LC3. **(D)** Protein aggregates and cytoplasmic components are targets of autophagy and can be non-selectively sequestered in the autophagosome. Selective autophagy targets specific cellular components for degradation. **(E)** Autophagosomes are formed after completion and closure of the phagosomal membrane with the facilitation of GABARAP and LC3 proteins. **(F)** Autophagosomes fuse with lysosomes and degradation initiates. mTOR, mammalian target of rapamycin; ULK, UNC-51-like kinase; UVRAG, UV radiation resistance-associated gene; ATG, autophagy-related; Ptdlns(3)P, phosphatidylinositol-3-phosphate; LC3, microtubule-associated protein light chain 3; GABARAP, gamma-aminobutyric acid type A receptor-associated protein (The picture was created with “BioRender.com”).

Under normal conditions, an effective mechanism for inducing autophagy is essential for organisms to adapt to stress or extracellular cues. An antagonistic interaction between the AMPK pathway and the mammalian target of rapamycin (mTOR) pathway regulates cellular autophagy levels, allowing cells to respond properly to extracellular variables. In general, mTOR1 inhibits autophagy while AMPK up-regulates it (Figure 1; Eshraghi et al., 2021).

Through distinct mechanisms, the nutrient-sensing mTOR1 detects both intralysosomal and cytosolic amino acids in eukaryotic cells (Condon and Sabatini, 2019). By recruiting and activating mTOR1, nutrients are utilized in anabolism, while autophagy and other catabolic pathways are inhibited (Hosokawa et al., 2009). As soon as mTORC1 is activated by Rheb, it phosphorylates ULK1 Ser757 and directly inactivates ULK1 (Figure 1A; Kim et al., 2011), preventing autophagy from the beginning during autophagosome biogenesis (Ganley et al., 2009).

As a result of energy deficiencies, nutrient limitation and stress signals, adenosine 5′-monophosphate (AMP)-activated protein kinase (AMPK) could be activated by LKB1 kinase, CaMKKβ, and ROS, among others. After activation, AMPK can phosphorylate and further activate TSC1/2 complex, which inhibits mTOR *via* upstream Rheb (Inoki et al., 2003). The cyclin-dependent kinase inhibitor p27kip1 is phosphorylated and activated by activated AMPK, which could further convert cells to a cell cycle arrest state. In response to energy insufficiency, p27kip1 prevents apoptosis and induces autophagy for cell survival (Liang et al., 2007).

### 2.2. Autophagy-related molecules

Autophagosome biogenesis begins with the activation of Unc51-like autophagy activating kinase 1 (ULK1) complex, which identifies the membrane upon which autophagosomes are formed (He and Klionsky, 2009; Nakatogawa, 2020). Beclin1 is a protein with only the BH3 domain, while BH3 proteins belong to the Bcl-2 family (Adams and Cory, 2001). A previous study indicated that the BH3 domain of beclin1 interacts with anti-apoptotic proteins such as Bcl-2/Bcl-XL and forms an inhibitory complex that inhibits autophagy induction (Walensky, 2006). Beclin1-PI3KC3 is a complex that plays a key role in autophagy initiation. The PI3KC3 complex abrogates lipid kinase activity and reduces autophagic flux (Sun et al., 2011). In contrast, as one of the several ATG core proteins, the ATG8/MAP1LC3 (microtubule-associated protein 1 light-chain 3, referred to as LC3) conjugate system is fundamental to extension and maturation of the autophagosome. In addition, cleaved LC3 coupled to phosphatidylethanolamine (PE) generates LC3-PE (LC-II) at levels known to correlate with the number of autophagosomes (Su et al., 2016). LC3 also acts as an adaptor protein during selective autophagy by binding to cargo receptors and recruiting selective cargo to autophagosomes (Rogov et al., 2014). Also controlled by LC3, LAP and LANDO play an important role in fungi and bacterial defection and the clearance of senescent or dead cells (Martinez et al., 2011).

### 2.3. Mechanisms of autophagy

#### 2.3.1. Autophagy

In order to initiate autophagy, the ULK1 complex is activated and recruited when AMPK is phosphorylated and mTORC1 is inhibited, forming and activating the ULK-Atg13-FIP200 complex and identifying the membrane where autophagosomes are formed (Turco et al., 2019; Vargas et al., 2019). In this way, the ULK complex activates PtdIn3K complexes such as UVRAG and ATG14L complexes. The former, characterized by UVRAG, is also involved in the co-fusion of autophagosomes and lysosomes. Whereas ATG14L, whose icon is the ATG14L protein, triggers the generation of PtdIns(3)P required for phagocyte nucleation and expansion (Itakura et al., 2008). In the following steps, ATG2 and ATG9 act together to transfer lipids across the membrane and re-equilibrate the membrane (Ghanbarpour et al., 2021). It is significant to note that the lipidation of ATG8 homologues induced by two ubiquitin-like conjugation systems is vital for their incorporation into the growing membrane (Lee and Lee, 2016). It is believed that ATG9 vesicles supply membrane sources, which also promote phagophore membrane elongation (Sawa-Makarska et al., 2020). The activated two ubiquitin-like binding systems promote the formation of ATG5-ATG12-ATG16L conjugate and complete LC3 lipidation with the assistance of WIPI (Figure 1C; Walczak and Martens, 2013).

ULK1 activation is coordinated with cargo consolidation during selective cargo capture, which can occur when mTOR is not regulated (Yamamoto et al., 2006; Lynch-Day and Klionsky, 2010), partly by adaptor proteins that facilitate cargo capture (He and Klionsky, 2009; Zaffagnini and Martens, 2016). In autophagosomes, protein aggregates and cytoplasmic components can be non-selectively immobilized. By utilization of adaptor proteins, such as P62, selective autophagy targets specific components of the cell for degradation (Eshraghi et al., 2021).

After formation, autophagosomes first fuse with endocytic structures to form amniotic bodies or directly with lysosomes with the assistance of other proteins (e.g., Rab7), eventually forming autolysosomes in which lysosomal hydrolases are activated by reduced pH to degrade cargo (Figure 1F; Ganesan and Cai, 2021).

#### 2.3.2. Mitophagy

Mitophagy is involved in the clearance of damaged mitochondria and the alleviation of the hyper-reactive neuroinflammation (Charmpilas et al., 2022). In AD, the accumulation of Aβ plaques and hyperphosphorylated tau increases and mitophagy processes decrease, thus enhancing neuroinflammation (Lautrup et al., 2019). Injured mitochondrial proliferation also leads to the activation of damage-associated molecular patterns (DAMPs) that act as pro-inflammatory molecules upon entry into the cytoplasm and exacerbate the neuroinflammation (Lerner et al., 2016).

There are three general types of mitophagy differentiated by generating process (Lemasters, 2014). Type 1 mitophagy is closely associated with nutrient recovery and cytoplasmic remodeling (Samuvel et al., 2022). In this process, beclin1/PI3K-mediated formation of phagophore coordinates with mitochondrial fission to isolate a mitochondrion into a mitophagosome and complete mitochondrial depolarization. The mitophagosome then fuses with the lysosomes and hydrolytically digests the encapsulated mitochondrion (Lemasters, 2014). Type 2 mitophagy proceeds similarly to type 1 mitophagy, except that the phagosomes are shaped differently and can form complete rings around damaged mitochondria (Lemasters, 2014). Type 3 mitophagy, also known as microfilaments, is associated with the formation of mitochondria-derived vesicles (MDVs), a PINK1/Parkin-dependent pathway (Lemasters, 2014; McLelland et al., 2014; Lemasters and Zhong, 2018). A few mitophagy receptors directly mediate mitophagy through protein-protein interactions (Giorgi et al., 2018), and these proteins form mitophagy receptors that target damaged mitochondria to autophagosomes for decomposition (Lemasters, 2014).

#### 2.3.3. LAP and LANDO

The LAP pathway utilizes components of the typical autophagic machinery, leading to the recruitment of LC3 to the phagosome *via* the PI3KC3 II complex and other autophagic proteins before fusion with the lysosome, resulting in a structure called the LAP-engaged phagosome (LAPosome). In contrast to canonical autophagy, LAP is not based on the AMPK-mTORC1-ULK1 axis and appears to not respond to intracellular stress sensing or nutrient status. In human microglia, the LAPosome is a long-lived cellular compartment. Therefore, this stability allows for slow degradation and long-term storage of antigen (Munz, 2018).

Endocytosis is an active process in which extracellular materials and plasma membrane components are engulfed in cell body. A number of physiological processes rely on endocytosis, including cell signaling and nutrient uptake. LANDO is a novel form of RME and receptor recycling, characterized by the association of LC3/GABARAP-family proteins with endosomal membranes (Heckmann et al., 2019). Endosomes mature from early to late multivesicular endosomes, which fuse with lysosomes to degrade cargo (Mullock et al., 1994). Recently, Heckmann et al. reported that primary microglia lacking the WD domain of ATG16L similarly lack LANDO and present a severe impairment of TREM2, TLR4, and CD36 recycling (Heckmann et al., 2020). LANDO-deficient microglia have a reduced ability to clear extracellular Aβ (Heckmann et al., 2020). LANDO-deficient mice develop AD-like symptoms and pathological changes of AD (Lv et al., 2013; Heckmann et al., 2020).

### 2.4. Microglia autophagy and neuroinflammation

Neuroinflammation refers to an inflammatory response within the CNS and PNS caused by pathological damage such as infection, trauma, and the accumulation of toxins. Neuroinflammation has long been recognized as a pathophysiological process associated with several NDDs (Niu et al., 2017; Ullah et al., 2017; Boyle et al., 2018). Recently, a growing body of evidence clearly indicates that autophagic activity is associated with neuroinflammation (Houtman et al., 2019). Microorganisms, damaged organelles and aggregates are generally considered to be the trigger of inflammatory signals. Autophagy removes these inflammatory signals and thus is regarded as an anti-inflammatory process in cells that induce autonomous inflammatory responses (Deretic et al., 2013; Deretic and Levine, 2018). Autophagic dysfunction and defective mitophagy may fail to limit the pro-inflammatory response in microglia, leading to the development of chronic inflammatory and NDDs (Sliter et al., 2018; Li W. H. et al., 2019).

M0-like and M2-like microglia are essentially anti-inflammatory, which may lead to an attenuated inflammatory response in the brain (Franco and Fernandez-Suarez, 2015). Depending on the internal environment, microglia can be polarized in both M1-like and M2-like phenotypes. M1-like microglia are usually induced by interferon-γ (IFN-γ), Aβ, and lipopolysaccharide (LPS) (Colonna and Butovsky, 2017). Although some microglia can differentiate into the M2-like phenotype, M1-like microglia activity predominates in AD. M2-like microglia are induced by anti-inflammatory cytokines such as IL-4 and IL-13 (Colonna and Butovsky, 2017). M1-like microglia release inflammatory cytokines and chemokines that induce inflammation and neurotoxicity, leading to inflammation and neuronal death in NDDs (Frank-Cannon et al., 2009). In contrast, alternative activation of M2-like microglia induce anti-inflammation and neuroprotection and is also responsible for absorption and removal of insoluble fibrous Aβ deposits (Heneka, 2017), contributing to tissue maintenance and repair in patients with AD (Colonna and Butovsky, 2017). Both types of microglia are involved in the pathogenesis of NDDs, and microglia in NDDs act as a double-edged sword (Tang and Le, 2016).

Autophagy in microglia interacts with neuroinflammation through multiple pathways, including PI3K/AKT, AMPK, mTOR, and cytokines (Zubova et al., 2022). Th1 cytokines are pro-inflammatory cytokines that activate autophagy, while Th2 cytokines are anti-inflammatory cytokines that inhibit autophagy (Torre et al., 2002; Wu et al., 2016). Evidence suggests that stimulation of autophagy can polarize microglia to the M2-like phenotype and inhibit subsequent inflammation (Jin et al., 2018). However, persistent neuroinflammation could inhibit microglia autophagy (Jin et al., 2018).

Mitochondrial production of ROS is a key upstream regulator of autophagy in NLRP3 inflammasomes and microglia (Yu and Lee, 2016). Excess ROS induces pro-inflammatory cytokine storms and DNA damage. Transcriptionally and post-transcriptionally, the various pathways through which ROS regulate autophagy also contribute to balance ROS levels and autophagy (Scherz-Shouval and Elazar, 2011). However, when this balance is disturbed, it can lead to detrimental consequences, for example, overloaded mitochondrial ROS can impair lysosomal function to block autophagic flux and drive microglia to M1-type polarization (Yuan et al., 2019).

## 3. Microglia autophagy and NDDs

**FIGURE 2:**
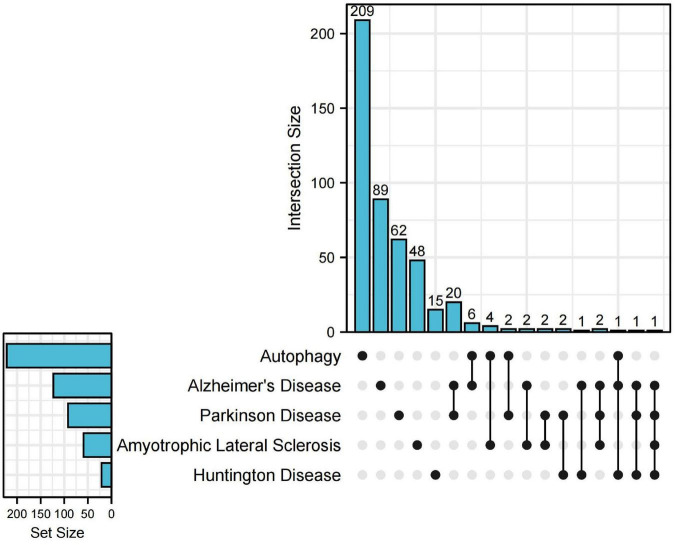
Upset plot for genes of neurodegenerative diseases (NDDs) and autophagy. The upset plot shows the autophagy-related genes in NDDs. The bar chart above represents the number of genes contained in each type of group. The bar chart at the bottom left represents the number of genes included in each type of NDDs and autophagy. The dotted line at the bottom right shows the genes contained in the group.

Heretofore, emerging evidence points to microglia autophagy defects affecting the onset and progression of NDDs. For example, the accumulation of Aβ in AD leads to neurotoxicity (Cohen and Paul, 1963). To investigate the relationship between microglia autophagy and NDDs at the genetic level, we searched the DisGeNET database^1^ for genes associated with AD, ALS, PD, and HD, respectively (with a score of 0.3 or more). Next, we looked for autophagy-related genes in the HADb database^2^, which intersect with genes related to the above four diseases. Upset plot was used for visualization by UpSetRR Package (Figure 2). By searching the disease database and the autophagy database for genes, we found that autophagy genes intersected with AD, PD, HD, and ALS, suggesting that these neurodegenerations may be associated with autophagy. Also, we were surprised to find that all four diseases were associated with the gene PPARGC1A. PPARGC1A (PPARG Coactivator 1 alpha) is a protein-coding gene. Diseases associated with PPARGC1A include Aging and ALS (Albani et al., 2016). This may indicate that these four diseases may share a common pathogenesis. Consequently, an in-depth investigation of the relationship between microglia autophagy and NDDs would benefit us to better understand these diseases.

### 3.1. Microglia autophagy in AD

Alzheimer’s disease refers to a remitting progressive memory loss and cognitive decline associated with neuropathology together with aging (Keren-Shaul et al., 2017). It was not until 1963 that Aβ plaques and neurofibrillary tangles were defined as key pathological features of AD by virtue of electron microscopy (Cohen and Paul, 1963). Emerging therapeutic approaches targeting Aβ and tau proteins themselves are failed at showing ideal efficacy in mitigating cognitive dysfunction in AD patients due to multifactorial influences (Nixon and Yang, 2011; Zeng et al., 2019). Specifically, the insufficient comprehension of combination therapies were based on the complex interactional nature of AD (Chong et al., 2018). Since Aβ theory was firstly proposed and demonstrated, it has gradually become the dominant theoretical basis for therapeutic innovation.

Recent investigation has emphasized that microglia, as crucial constituents for neural homeostasis (Hindle, 2010; Del Rey et al., 2018), also acts as the key part in the mechanistic theory of AD. Microglia attributes to the integrality and survival of both neurons and microenvironment (Tremblay et al., 2011). While in pathological conditions, microglia responses rapidly and results in diversified outcomes, one of which is that microglia inflammation and neuronal apoptosis can be stimulated through the Junn-terminal kinase (JNK) signaling pathway (Bai et al., 2010; Houtman et al., 2019). Particularly, abnormal neuroinflammation and subsequent neurodegeneration of the CNS may be attributed to a specific type of it (Keren-Shaul et al., 2017; Li et al., 2018). Therefore, microglia and neuroinflammation are regarded as the core of the pathological mechanism.

**FIGURE 3:**
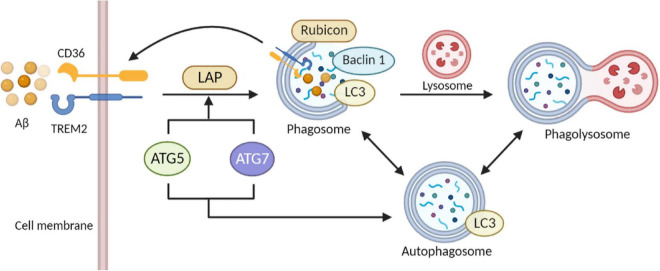
Role of autophagy and phagocytosis in microglia clearance of Aβ. The close link between autophagy and phagocytosis makes it a therapeutic target for AD. Activation of ULK1 initiates microglia autophagy. Interaction of Aβ with TREM2 and CD36 on microglia membranes initiates phagocytosis. Subsequently, ATG 5 and ATG 7 activity mediates the recruitment of LC3 to form single-membrane phagosomes. Transfer of Beclin1 and LC3 proteins to phagosomes enhances the fusion of the phagosome-lysosome system. The interaction between autophagy and phagocytosis elevates the efficiency of microglial phagocytosis and facilitates autophagy in eliminating extracellular cargoes including Aβ aggregates (The picture was created with “BioRender.com”).

Proved by *in vivo* experiments, autophagy impairment has been confirmed as the major contributor to brain dysfunction and NDDs by attenuating the clearance of Aβ (Broz and Dixit, 2016; Zeng et al., 2019). As for the molecular mechanism, mutations in the various related genes may provide a novel sight of genetic causes. A previous study finds mutations in the PS1 gene were closely related to the disruption of lysosomal acidification/proteolysis through fibroblasts in AD patients (Nixon and Yang, 2011). Also, autophagosome formation in induced pluripotent stem cells (iPSCs) is impaired while depleting PS1. At the same time, some autophagy-related gene expression was down-regulated after gama-secretase independent ERK/CREB signaling pathway-was inhibited (Chong et al., 2018). Coincidentally, interruption of autophagy has been shown in other AD mouse models with over-expressed mutant APP, potentially based on the toxic effect of β-secretase lysis carboxyl end fragments (βCTF) that can injure lysosomes (Yang et al., 2011). Besides, recent investigations prove that mutations in the ATG gene can modulate the neurodegenerative phenotype of mice. In experimental models, conditional knockdown of ATG5 and ATG7 was observed to have an accumulation of cytoplasmic inclusion bodies such as polyubiquitinated proteins, eventually leading to neuronal death (Hara et al., 2006; Komatsu et al., 2006). Autophagy enhancement in mouse models of NDDs is reported with an evident decline of cytoplasmic inclusion bodies and improvement of disease phenotypes. For example, in the latest study, PPARA-mediated autophagy activation and CD36 and TREM2 receptor-mediated Aβ uptake reduced cognitive decline in AD (Figure 3; Luo et al., 2020).

Nevertheless, impaired microglia autophagy up-regulated IL-1 and IL-18 expression in microglia causing increased cytoplasmic levels of inflammasome and NLRP3 and CASP1/Caspase1 cleavage (Houtman et al., 2019), which accelerates AD progression.

In summary, microglia autophagy deficiency plays an essential role in the pathology of AD *via* regulating the secretion of inflammatory cytokines or other subcellular substances accompanied with abnormal accumulation of Aβ. Enhancement of autophagy may provide a novel mechanistic target by reducing the number of cytoplasmic inclusion bodies.

### 3.2. Microglia autophagy in PD

Parkinson’s disease was regarded as one of the most grievous movement disorders for its pathologic features, including tremors and postural instability. PD has a prevalence of nearly 1% in people during the past decades while 10% of cases are of genetic origins and other idiopathic sources (Toulouse and Sullivan, 2008; Su et al., 2016). Hereditary factors together with exposure to environmental toxins mainly contribute to chronic and progressive deficiency of dopaminergic neurons in the dense part of the substantia nigra (SN) in patients with PD (Forno, 1996). Mutations at 23 loci including LRRK2 (PARK8), SNCA (PARK1), PINK-1 (PARK6), and PRKN (PARK2), etc. are further investigated by numerous studies over the past 20 years (Klein and Westenberger, 2012; Del Rey et al., 2018). Meantime dopaminergic neuronal death, and chronic neuritis are identified as a part of pathological features of PD (Poewe et al., 2017).

A great body of investigation highlights that microglia-induced chronic neuroinflammation is highly associated with the onset and progression of PD (Schiess et al., 2010; Pagano et al., 2016). Inflammasomes are subcellular macromolecular complexes that are assembled and regulated by inflammatory proteases from the caspase family (Schwarcz et al., 2010; Ochaba et al., 2014). Since NLRP3 (NLR family, containing 3 pyrin domain) inflammasomes are proved involved in the pathological progression in both human and mouse models (Han et al., 2019), microglia may also serve as the key factor in the whole mechanism.

Multiple evidence suggests that autophagic dysfunction, associated with inflammation-induced disorders, contributes to the pathogenesis of neurodegenerative changes in mouse models (Komatsu et al., 2006; El-Khider and McDonald, 2016). However, the majority of studies focus on neuronal cells instead of microglia. For current investigations, more attentions are paid to the role played by microglia-induced inflammation of the pathological basis for PD. According to the study on drosophila, Manf (neurotrophic factor derived from midbrain astrocytes) is regulated by autophagy in immune cells, resembling microglia in vertebrates (Burrell et al., 2016). Furthermore, microglia autophagy in mammalian cells underpins microglia activation *in vitro*. Deficiency of microglia autophagy leads to up-regulation of pro-inflammatory cytokines together with elevated activation of M1 microglia (Sierra et al., 2013; Su et al., 2016). Further investigation shows that microglia autophagy defects mechanically activate NLRP3 inflammasomes *via* PDE10A-cAMP signaling and exacerbate dopaminergic neurodegenerative degeneration and neuroinflammation. In addition, the loss of microglia ATG5 is shown to result in PD-like symptoms in mice, including cognitive deficiency, motor coordination disorders as well as decreased striatal dopamine levels (Cheng et al., 2020; Qin et al., 2021).

### 3.3. Microglia autophagy in HD

Huntington’s disease, inherited in an autosomal-dominant manner (Ross and Tabrizi, 2011), is defined as a distinctive neurodegenerative disease and characterized by chorea, dystonia and incoordination (Walker, 2007). The its onset and progression of HD have been proved to be associated with the disorder of protein misfolding. Discovered 29 years ago, Huntingtin protein (HTT) is considered as the major contributor in HD, revealing the mechanistic method of the pathogenesis (Lorente Pons et al., 2020). The expansion of CAG trinucleotide repeats in the first exon of HTT, thus encoding an expanded polyglutamine tract in mutant Huntingtin protein (mHTT) and contributing to its incorrect conformation and aggregation in neurons (Ross and Tabrizi, 2011).

Emerging evidence suggest that microglia secrete cytokines while stimulated by abnormal protein, such as mHTT, causing further microglial activation, neuronal dysfunction, and death (Koch et al., 2016). In agreement with findings in PET imaging (Pagano et al., 2016), *in vitro* research, and post-mortem studies, microglia activation is associated with prodromal stage and subclinical progression of HD (Tai et al., 2007). Indeed, potential therapeutics could be identified through studies of the mechanistic role played by microglia.

Moreover, previous published data indicate that wild-type HTT acts as an ATG11-like scaffold protein for selective autophagy (Ochaba et al., 2014), while relatively empty autophagosomes are observed in mHTT-existing cells (Martinez-Vicente et al., 2010). Autophagy, participating as a self-degradative process (Glick et al., 2010), contributes to numerous biochemical processes including cellular homeostasis maintenance and deposition of misfolded protein, damaged mitochondria, reactive oxygen species in HD (Munz, 2016). Of note, nucleotide-binding oligomerization domain-, leucine-rich repeat- and pyrin domain-containing 3 (NLRP3), a widely studied inflammasome, abundantly expressed in microglia and triggered neuroinflammation in HD. Nevertheless, recent data shows microglial autophagy is highly associated with the stability of brain homeostasis and negative regulation of NLPR3 inflammasome-regulated neuroinflammation (Wu et al., 2021). As such, inducers of microglia autophagy could be identified as a potential HD treatment.

### 3.4. Microglia autophagy in ALS

Amyotrophic lateral sclerosis is a debilitating neurodegenerative disease characterized by the loss of motor neurons, paralysis together with cognitive changes ranging from mild deficiency to severe FTD (Talbot et al., 1995).

Importantly, mutations in autophagy-related genes are specifically tuned in ALS. Mutations of critical genes (TREM2, C9Orf72, GRN, and PFN1, etc.) are responsible for both altering the activation of phagocytes together with inflammatory pathways and interfering with microglia functions in patients with ALS (Haukedal and Freude, 2019; Jin et al., 2021).

Normally, autophagy was proved to promote the anti-inflammatory phenotype of microglia *via* blocking the secretion of pro-inflammatory cytokines as well as inflammatory vesicles. Inversely, autophagic deficiency contributes to successive abnormal microglia functioning and chronic neuroinflammation and degradation (Plaza-Zabala et al., 2017). Furthermore, microglia are indispensable for eliminating accumulated pro-inflammatory STING (interferon gene stimulating factor) protein and other metabolites (McCauley et al., 2020), thus participating in neural homeostasis maintenance and pathology of NDDs. All these findings emphasize the co-interaction between microglia autophagy, neuroinflammation, and ALS (Crisan et al., 2011).

Remarkably, studies show that stimulation for autophagy holds great promise for SOD1-related ALS treatment since the oligomerization of SOD1 inclusions is proven to delay the onset and progression of ALS (Plaza-Zabala et al., 2017). Whereas, SOSTM1-positive cytoplasmic inclusions, as an essential part of the clearance of polyubiquitinated mitochondria, are identified in the majority of both ALS patients and other neurological disorders (Lorente Pons et al., 2020; Kuusisto et al., 2001). Evidence shows that overexpressed SQSTM1 promotes the progression of ALS through attenuating autophagic activation and/or degradation in LC3-II positive autophagic vesicles. Interestingly, there is a positive feedback loop consisting of SQSTM1, KEAP1, and NRF2 that can significantly enhance selective autophagy, contributing to the deposition of damaged organelles (Bellezza et al., 2018). Together, growing attention is paid to microglia autophagy and its characteristics associated with ALS like inflammatory vesicle activation and protein clearance.

## 4. Therapeutics for NDDs

### 4.1. Clinically used drugs

A number of drugs have been approved so far to treat age-related NDDs. For instance, AD is treated with cholinesterase inhibitors, including tacrine, donepezil, rivastigmine, galantamine, para-amino-benzoic acid, flavonoids, and pyrrolo-isoxazole analogues (Anand and Singh, 2013). AChE inhibitors enhance neuronal function by increasing the concentration of acetylcholine *via* inhibiting the biological activity of acetylcholinesterase (Tabet, 2006). Notably, due to its hepatotoxicity and adverse side effects at high doses, tacrine was discontinued in an early time (Watkins et al., 1994). Levodopa is an extremely effective drug for treating PD. Nevertheless, prolonged treatment with levodopa can lead to motor complications, such as fluctuations in clinical response (Espay et al., 2018). HD is treated symptomatically with drugs such as haloperidol, endorphin, thiopride, and chlorpromazine, which block dopamine receptors. Moreover, ALS was postulated to be delayed when treated with by rilozule, a glutamatergic neurotransmission inhibitor (Jaiswal, 2019), but the exact mechanism is not known.

There are, however, no drugs or treatments available *via* modulating microglia autophagy to treat age-related NDDs. By summarizing recent studies on potential drugs that target microglia autophagy, we provide promising directions for subsequent investigations in drug managements of NDDs.

### 4.2. Potential drugs and therapeutics

#### 4.2.1. Therapeutics targeting microglia autophagy or phagocytosis

It has been shown that fluoxetine promotes phagocytosis in microglia and phagocytosis of amyloid β1-42-GFP increased with fluoxetine pretreatment, suggesting that fluoxetine may promote clearance of Aβ by microglia. In microglia, fluoxetine induces autophagy by increasing the accumulation of the autophagic protein LC3-II. According to immunofluorescence observations, microglia treated with fluoxetine exhibit a significant increase in LC3 puncta, indicating fluoxetine induces autophagy and increases autophagic flux (Park et al., 2021). Recently, it has been reported that the small molecule kaempferol (Ka) promotes cytophagy/autophagy in microglia by increasing MAP1LC3B-II expression level, resulting in decreased NLRP3 protein expression and inactivation of NLRP3 inflammatory vesicles. In addition, Ka promotes neuroinflammation suppression *via* ubiquitination and autophagy and offers the potential as a therapeutic strategy for PD and other NDDs (Han et al., 2019).

Some studies have also investigated potential drugs targeting serotonin receptors. 5HT2A receptor (5HT2AR) is a subtype of the 5HT2 receptor, which are widely expressed throughout the CNS and play a variety of roles in the brain. Desloratadine (DLT) is a second-generation H1 antagonist that selectively inhibits 5HT2AR and stimulates autophagy through the 5HT2AR/cAMP/PKA/CREB/Sirt1 pathway, which inhibits neuroinflammatory responses, activates glucocorticoid receptor nuclear translocation, and further induces TLR2 and TLR4 transcription in response to microglial phagocytosis (Cerminara et al., 2013). Immunofluorescence imaging of the autophagy marker protein LC3 showed that DLT stimulated microglia autophagy as well in AD mouse model (Lu et al., 2021).

Parkinson’s disease is characterized by the accumulation of Lewy vesicles that contain fibrillogenic α-synuclein(α-syn). Toll-like receptors (TLRs), especially TLR2, are increased in PD brains, and pathological accumulation of α-syn is closely linked to TLR2 activation in PD brains. According to previous study (Dzamko et al., 2017), rapamycin promotes cellular autophagy and inhibits TLR2, preventing the TLR2-mediated increase of synuclein, suggesting that activation of autophagy can relieve synuclein accumulation. Besides, metformin, an oral hypoglycemic agent commonly used in treating diabetics, also prevented DA neuronal degeneration, attenuated α-syn accumulation, and enhanced neuronal autophagy in substantia nigra compacta (SNc). Of importance, the enhanced autophagy may be related to an increase in phosphorylation levels of Thr172 in the active site of AMPK in the midbrain (Lu et al., 2016). The role of metformin in enhancing microglia autophagy, however, remains to be determined.

Research on plant extracts is also promising. Extracts from Withania somnifera (WS; also known as Indian ginseng) have protective effects on the nervous system, and these effects may be caused by a decreased activation of the NF-B pathway in microglia. In mouse model, it reduced spinal cord inflammation and could be utilized in treating ALS (Dutta et al., 2018). Interestingly withaferin A in WS extract induces autophagy (Hahm and Singh, 2013) *via* up-regulating LC3-II protein and down-regulating p62 protein, which promotes autophagosome formation (Dutta et al., 2018).

In addition achyranthes bidentate polypeptide fraction k (ABPPk) (Ge et al., 2021), catechins (Sebastiani et al., 2021), Dendrobium nobile Lindl alkaloid (Li D. D. et al., 2022), memit (a prodrug of memantine) and tyrosine kinase inhibitors have all been shown to directly or indirectly influence the regulation of microglia autophagy and have a wide range of prospects in the treatment of NDDs (Javidnia et al., 2017; Sestito et al., 2019). Notably, ABPPk regulates neuroinflammation and alleviates neurotoxicity by restoring autophagy in damaged microglia, promoting M2-like-phenotype polarization (Ge et al., 2021). Most of those investigations are *in vivo* or *in vitro* studies, and a few have been used in clinical trials (Table 1; Gu et al., 2014; Lu et al., 2016; Alcocer-Gomez et al., 2017; Dutta et al., 2018; Holczer et al., 2018; Sestito et al., 2019; Shu et al., 2019; Lv et al., 2020; Ge et al., 2021; Joshi et al., 2021; Kodali et al., 2021; Lu et al., 2021; Park et al., 2021).

**TABLE 1:** Microglia autophagy-targeted tests and outcomes.

Drugs	Type of study	Methodology	Major outcomes	References
Withania somnifera (WS)	Transgenic (Tg) mouse model of ALS *N* = 25 (WS treated) *N* = 25 (Vehicle treated)	Identification of clinical symptoms; Motor performance test; IHC; Immunoprecipitation for misfolded protein; Immunoblot analysis; Cytokine array	WS treatment induces autophagy of microglia since LC3-II was found significantly highly expressed in the experimental group (WS-treated)	Dutta et al., 2018
	*In vitro* model (HT22 cell) Mouse of HD for *in vivo* model (all treated with 1mg/kg drug)	Behavioral studies; Striatal volume measurement; Immunostaining and aggregate counting; Dot blot assay and immunoblotting; Semi-quantitative PCR	WA was shown to attenuate proteasomal dysfunction and induction of autophagy	Joshi et al., 2021
Epigallocatechin-3-gallate (EGCG)	*In vitro* model	SDS-PAGE and WB; RNA Interference; RNA Extraction and Real-Time PCR; Cell Viability Assays	EGCG promotes autophagy through mTOR-dependent and PKA-independent molecular pathways	Holczer et al., 2018
	Wistar rat for *in vivo* model *N* = 10 (control group) *N* = 10 (CUMS-treated) *N* = 10 (CUMS + CQ) *N* = 10 (CUMS + EGCG) *N* = 10 (CUMS + EGCG + CQ) *N* = 10 (vehicle + CUMS)	Plasma crticosterone concentration assessment; MWM test; HE staining; TUNEL assay/TEM; WB/ELISA	EGCG restores CUMS-impaired autophagic flux in rat CA1 region	Gu et al., 2014
Desloratadine (DLT)	AD-like pathology of APP/PS1 mouse for *in vivo* model	IHC; IF; WB/ELISA/RT-PCR of brain tissue	DLT regulates macroglia autophagy through 5HT2AR/cAMP/PKA/CREB/Sirt1 pathway	Lu et al., 2021
Metformin	*In vitro* model; *In vivo* model (1-methyl-4-phenyl-1,2,3,6-tetrahydropyridine plus probenecid-induced mouse model for PD) Group1 (saline-treated), Group2 (MPTP/p-treated), Group3 [MPTP/p + metformin (MET)-treated], Group4 (MET alone treated)	Rotarod Test; High Performance Liquid Chromatography Analysis; WB/IHC/qt-PCR; Assay of MTT Conversion; Assay of Lactate Dehydrogenase (LDH) Release; Flow Cytometric Analyses; Detection of Intracellular ROS	Metformin (2mM) induces 3-II-mediated macroglia autophagy relied on AMP-activated protein kinase and microtubule-associated protein 1 light chain.	Lu et al., 2016
	Male C57BL6/J mice for *in vivo* model Group 1 (young and 18-month-old mice), *n* = 11–12, for neurobehavioral test Group 2 (control aged), *n* = 12 Group 3 (aged-MET treated), *n* = 12	PST/OLT/novel object recognition test; Tissue processing, IHC, quantification of various cells; Morphometric analysis; Immunofluorescence staining; Confocal microscopic analyses; Measurement of Syn +, PSD95 +, and p62 + structures; Biochemical assays	Long-term MET treatment induced significant reduction of p62 + structures in the CA3 pyramidal neurons, and enhancement of autophagy.	Kodali et al., 2021
Fluoxetine	*In vitro* model	Microglia isolation; Nitric oxide measurement and cell viability assay; Reverse Transcription–Polymerase Chain Reaction; WB/Phagocytosis Assay/IF Assay	Fluoxetine contributes to LC3-II accumulation thus closely associated with autophagy initiation.	Park et al., 2021
	Clinical trial *N* = 20 (MDD patients without treatment) *N* = 15–21 (MDD patients treated with different drugs for parallel)	Cells isolation; Test for IL-1β/18 levels; RT-qPCR	Genes involved in autophagy were shown reduced in Fluoxetine-treated group	Alcocer-Gomez et al., 2017
	Male C57/BL6J mice for *in vivo* model Group 1: suitable environment for control Group 2: stressed animals	Sucrose preference test; FST/TST; Corticosterone content determination; TEM/WB analysis; mTagRFP-Wasabi-LC3 plasmid transfection; puncta counting for fluorescence; Immunofluorescent staining; Flow cytometry assay; Mitochondria and lysosome staining; ROS detection; Cell viability assay	By inducing fusion of autophagosomes with lysosomes, fluoxetine promotes the autophagic flux in astrocytes.	Shu et al., 2019
Memantine prodrug (Memit)	*In vitro* model	ROS production; Glioma cell culture; WB; [3H]MK-801 Binding Assay; Thioflavin T fluorescence assay; amperometric assay	Expression of macroglia autophagy-related protein, such as LC3-II, p62, etc. were detected to be enhanced in Memit-treated group	Sestito et al., 2019
Achyranthes bidentate polypeptide fraction k (ABPPk)	*In vitro* model; *In vivo* model (C57BL/6 mice) *N* = 15 (sham group), *N* = 15 (AβOs group), *N* = 15 (ABPPk group)	BV2 Microglia-Conditioned Media System; qPCR/Immunocytochemistry; Open Field Test/Morris Water Maze Test; Fluoro-Jade C Staining; Enzyme-Linked Immunosorbent Assay; WB	ABPPk influences M1/M2 polarization through restoring the AβOs-damaged autophagy in microglia, which promoting M2-phenotype polarization while inhibiting M1-phenotype polarization of macroglia	Ge et al., 2021
Dendrobium nobile Lindl alkaloid	*In vivo* model [senescence-accelerated mouse-prone 8 (SAMP8) mice] Group 1 (SAMP8 model), Group 2 (DNLA low-dose), Group 3 (background control with aged-matched SMAP1 mice)	Y maze; Open field test; Rotarod; Hematoxylin-eosin staining; Nissl staining; SA-β-gal staining	DNLA and metformin enhanced autophagy activity of macroglia by enhancing the expressions of LC3-II, Beclin1, etc.	Lv et al., 2020

#### 4.2.2. Therapeutics targeting microglia mitophagy

Several NDDs, including PD, may be associated with the accumulation of damaged mitochondria (Rambold and Lippincott-Schwartz, 2011). Thus, modulating mitophagy is crucial to NDD management. As an intrinsic response to control mitochondrial quality, mitophagy is a form of cellular autophagy that selectively removes defective mitochondria. By blocking the release of mtDNA and mtROS from damaged mitochondria and limiting the activation of NLRP3 inflammatory vesicles, mitophagy prevents neuroinflammation from the onset (Ding et al., 2022; Qiu J. R. et al., 2022; Qiu W. Q. et al., 2022), mitophagy deficits and oxidative stress induced by cellular energy deficit. Are responsible for causing NDDs such as AD and PD (Rambold and Lippincott-Schwartz, 2011; Chen et al., 2021). Controversially, mitochondrial ATP deficiency may also induce autophagy *via* mTOR/AMPK activation (Rambold and Lippincott-Schwartz, 2011).

According to earlier studies, melatonin reduces AD-like pathology through the restoration of autophagic flux and by promoting mitophagy (Pandi-Perumal et al., 2013; Ganie et al., 2016). Additionally, melatonin significantly improved cognition and reduced Aβ deposition in AD mouse model (Luengo et al., 2019). Melatonin has been shown to reverse AD-related protein expression, including Trem2, Gfap, Syt11, HK2, and Mcoln1. Those proteins have extensive biological functions during the process of protein autophosphorylation, mitochondrial autophagy phagocytosis, and innate immunity (Chen et al., 2021). For instance, Mucolipin-1 (Mcoln1) is involved in phagosomal-lysosomal fusion. Expressions of proteins (Fnbp1l, Sirt2, ATP5IF1, ATG2b, and Mcoln1) involved in mitophagy have been demonstrated to be enhanced by melatonin, which positively regulate lysosome-mediated degradation or intranuclear endosome transportation (Samie et al., 2013). Moreover, the melatonin ameliorated mitophagy deficits, improved mitochondrial function in the hippocampus, and reduced Aβ deposition in the hippocampus of AD mouse model (Chen et al., 2021). Therefore, melatonin might be a potential therapeutic agent for AD.

NLRP3 inflammasome activation and neuroinflammation are associated with NDDs (Holbrook et al., 2021). By up-regulating SHP-2 in BV-2 cells, polygala saponins (PSS) activated AMPK/mTOR and PINK1/parkin signaling pathways, which led to mitophagy. PSS significantly inhibited NLRP3 inflammasome activation induced by A53T-α-synuclein or Q74 in microglia, suggesting mitophagy may surpress inflammasome activation (Qiu W. Q. et al., 2022).

An original study found that capsaicin attenuated mitochondrial depolarization and rescued mitophagy defects in preformed fiber (PFF)-tolerant microglia, while autophagic flux was up-regulated (Lu et al., 2022). Capsaicin might contribute to the degradation of α-syn in PD or Aβ in AD.

#### 4.2.3. Therapeutics targeting neuroinflammation

Microglia activation is associated in different neuroinflammatory pathologies of the brain (Chagas et al., 2020). Therefore, manipulation of microglia activation and release of inflammatory cytokines can largely control neuroinflammation and thus treat NDDs. For instance, lithium reduces the release of pro-inflammatory mediators from microglia *in vitro*, while enhance production of IL-10, an anti-inflammatory cytokine (Fabrizi et al., 2017). However, no studies have explored the precise molecular mechanism of lithium, which was postulated to be associated with inositol monophosphatase, phosphoglucomutase, and GSK-3 (O’Brien and Klein, 2009).

Parkinson’s disease models could be established by inducing mitochondrial dysfunction and inflammation with rotenone (Johnson and Bobrovskaya, 2015). Pre-treatment with Pyrroloquinoline Quinone (PQQ) significantly blocked rotenone-induced up-regulation of pro-inflammatory factors such as interleukin-1β (IL-1β), IL-6 and tumor necrosis factor-α (TNF-α) in a dose-dependent manner, and also significantly reduced NO production (Zhang et al., 2020). It is suggested that PQQ may suppress cytokine storms in neural tissues by inhibiting rotenone-induced inflammation in BV2 microglia. PQQ may also induce autophagy in BV2 microglia treated with rotenone (Zhang et al., 2020). As a neuroprotective compound derived from cornus fruits, loganin is effective in aliviating inflammation inhibiting excessive autophagy in PD mouse model induced by 1-methyl-4-phenyl-1,2,3,6-tetrahydropyridine (MPTP) (Xu et al., 2017).

Extended and amplified microglia activation may also contribute to Aβ clearance. *In vitro*, spermidine increased the expression of the autophagy-associated gene ETS2, as well as microglia cluster 2, both of which enhance phagocytosis and degradation of Aβ. Furthermore, spermidine may have a direct inhibition on Aβ-induced neuroinflammation by reducing NF-κB phosphorylation levels and down-regulating inflammatory cytokine-related genes (Freitag et al., 2022).

A previous study reported that HD mice displayed improved motor functions when using selective mGluR2/3 agonists with improved to reduce mutant huntingtin aggregate formation, neuronal cell death, and microglia activation in the striatum. Similarly, activating the glycogen synthase kinase 3β-dependent autophagic pathway in male HD mice reduces mutant huntingtin aggregates (Li S. H. et al., 2021). Moreover, β-Caryophyllene (BCP) (Borgonetti et al., 2022) and echinocysticacid (EA) (He et al., 2022) also inhibit microglia-mediated neuroinflammation by inhibiting the release of pro-inflammatory cytokines and promoting the release of anti-inflammatory cytokines from microglia.

#### 4.2.4. Potential nanomedicines

As a state-of-the-art therapeutic strategy, nanotechnology offers an array of possibilities by enabling interconnected platforms to solve unmet needs and problems (Malviya et al., 2021). Nanomedicines’ nanoscale size (1–100 nm) and large surface area make them ideal platforms for accessing biological targets and interacting with cells and tissues precisely (Sahoo et al., 2007). Therefore, the application of nanomedicines in age-related NDDs holds great promise.

**FIGURE 4:**
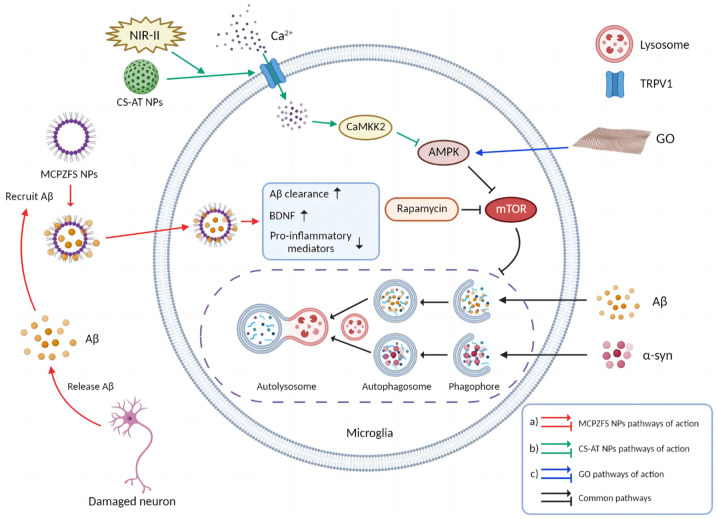
Molecular mechanisms of nanomedicines. **(A)** MCPZFS NPs recruited Aβ and were endocytosed into microglia. After the dysfunctional microglia were restored, secretion of pro-inflammatory mediators was reduced, while phagocytosis of microglia, production of BDNF, and Aβ clearance were enhanced. **(B)** CS-AT NPs targeted microglia and opened their surface TRPV1 channels after the second near infrared (NIR-II) laser irradiation, leading to Ca2 + influx, activations of ATG5 and Ca2 + /CaMKK2/AMPK/mTOR signaling pathways, enhanced autophagy, phagocytosis and degradation of α-syn. **(C)** After GO entered microglia, it activated AMPK and inhibited mTOR pathway. GO-activated autophagy is analogous to rapamycin-activated autophagy (The picture was created with “BioRender.com”).

According to a recent report, zwitterionic poly (carboxybetaine) (PCB)-based nanoparticles (MCPZFS NPs) targeting the normalization of dysfunctional microglia and Aβ recruitment is established for the treatment of AD. MCPZFS NPs significantly reduced the release of pro-inflammatory cytokines such as TNF-α, IL-2β, IFN-γ, and ROS from microglia. Besides enhancing phagocytosis of Aβ, PCB also promotes degradation of Aβ. At the presence of MCPZFS NPs, the Aβ degradation shifts from the conventional lysosomal/autophagy to the proteasomal pathway (Liu et al., 2020). MCPZFS NPs have the ability to recruit Aβ into microglia and enhance Aβ phagocytosis, which may potentially contribute to non-covalent interaction between PCB and Aβ (Figure 4A). There is a possibility that PCB-based nanomaterials could be used as zwitterionic drugs to treat AD (Malviya et al., 2021).

It is worth noting that as a nanomaterial, graphene oxide (GO) inhibits the mTOR signaling pathway by activating AMPK, leading to microglia activation and neuronal autophagy (Figure 4C). Moreover, with the inhibition of microglia autophagy, GO promoted microglia-mediated Aβ phagocytosis. GO was not only non-cytotoxic to microglia and neurons, but also reduced the toxicity of Aβ *via* enhancing microglia clearance (Li et al., 2020). These findings provide new theoretical rationales for the treatment of NDDs.

Parkinson’s disease is characterized by the accumulation of Lewy bodies with fibrillogenic α-syn as the main component in neurons (Frigerio et al., 2011). In PD brain, toll-like receptors (TLRs), particularly TLR2, are increased, and pathological accumulation of α-syn is closely associated with TLR2 expression. Rapamycin promotes cellular autophagy and inhibits TLR2 signaling pathway which further blocks TLR2-mediated elevation of α-syn (Dzamko et al., 2017). Cu2-xSe-anti-TRPV1 nanoparticles (CS-AT NPs) could assist opening microglial surface TRPV1 channels under secondary near-infrared (NIR-II) laser irradiation and further induces Ca2 + infux with activation of ATG5 and Ca2 + /CaMKK2/AMPK/mTOR signaling pathways, which promotes phagocytosis and degradation of α-syn in PD (Figure 4B; Yuan et al., 2022).

Recently, a Prussian blue/polyamidoamine dendrimer/Angiopep-2 (PPA) nanoparticles was developed and exhibited excellent blood-brain barrier permeability and ROS scavenging ability. PPA could contribute to the restoration of mitochondrial function in microglia, inhibit excessive mitophagy and thus prevent excessive microglia activation in AD mouse model (Zhong et al., 2022). With its excellent permeability, PPA offers huge potential for nanomedicine research in the filed of NDD treatment.

Many conventional drugs have diverse inherent drawbacks, such as short blood half-life and poor blood-brain barrier (BBB) penetrability, which severely limit their efficacy in NDD treatment (Moscariello et al., 2018). It has been reported that NPs might be free of these drawbacks. For instance, GO, a derivative of graphene, has abundant hydrophilic groups and a high stability in aqueous dispersions (Singh et al., 2018). Moreover, the dimensions of GO nanosheets satisfy the dimensional requirements for crossing the BBB, proving their potential as drug delivery carriers (Li et al., 2020). Notably, the ability of Prussian blue to effectively scavenge ROS suggests its neuroprotective effect against NDDs, despite its poor permeability crossing the BBB. PAMAM dendrimer (PAMAM-G4, ∼4 nm), as a kind of NPs, can cross the damaged BBB after systemic administration and selectively target highly active microglia in NDD models (Zhong et al., 2022). Importantly, nanomedicines also have their own limitations, for example, MCPZFS NPs are buffered in a wide pH range (Liu et al., 2020), suggesting that the molecular form of MCPZFS NPs may change as the pH changes. Moreover, CS-AT NPs caused the opening of TRPV1 channels on the surface of cells other than microglia, and Ca^2+^ inward flow in other neural cells may proceed their activation.

#### 4.2.5. Potential targets for microglia autophagy

In recent years, promising targets closely related to the regulation of microglia autophagy have been explored by several teams through wet-lab experiments and clinical trials (Table 2; Yan et al., 2017; Li Y. et al., 2019; Yin et al., 2019; Lim et al., 2021; Chung et al., 2022). Evidence shows miRNAs are important regulators of autophagy (Xu et al., 2012). Deficits of Mir223 inhibit pathogenic demyelination in the CNS *via* enhancing autophagy in experimental autoimmune encephalomyelitis mouse model (Li Y. et al., 2019). Indeed, Mir223 regulates microglia autophagy by targeting the autophagy-related ATG16l1 gene, and this regulation is independent of the BCL2 and PPARG pathway. Hence, knockdown of Mir223 or inhibition of endogenous Mir223 increased autophagy in microglia and resting microglia (Li Y. et al., 2019). Therefore, Mir223 could be a potent target for the treatment of NDDs by improving/limiting uncontrolled or potentially harmful autophagic activity in microglia.

**TABLE 2:** Tests to explore targets for regulating microglia autophagy.

Potential target	Type of study and sample size	Method	Current findings	References
Clk1 (coq7)	*In vitro* model; MPTP-induced mouse model of PD, randomly and equally divided into three groups (1) saline, (2) MPTP, (3) metformin + MPTP	Plasmid and LV MTT assay IF/WB/q-PCR ADP/ATP Ratio Assay Rotarod test/pole test/IHC	Clk1 directly regulates autophagy of dopaminergic neurons via the AMPK/Mtorc1 pathway, attenuating microglia-mediated inflammation.	Yan et al., 2017
Maresin 1	*In vitro* model; C57BL/6 mice for *in vivo* model *N* = 10 (PBS and solvent of MaR1), *N* = 10 (Aβ42 and solvent of MaR1), *N* = 10 (PBS and MaR1 solution), *N* = 10 (Aβ42 and MaR1 solution)	MWM Test IHC FJB staining CBA/ELISA/WB	MaR1 enhances autophagy by inhibiting Aβ42-induced mTOR pathway.	Yin et al., 2019
Neuronal TLR2/4 pathway	Human study (516 PD patients and 513 healthy controls); *In vitro* model	ELISA kit for plasma samples; Transwell coculture; Real-Time Live Cell Confocal Imaging; IF labeling/CTCF; Cytokines measurements; qt-PCR/WB	The activation of neuronal TLR2/4 perturb the autophagy flux via p38/JHK pathway.	Chung et al., 2022
Human neural crest-derived nasal turbinate stem cells (hNTSCs)	*In vitro* model; Human AβPP and PS1 mutant mouse for *in vivo* model *N* = 15 (wild-type with PBS) *N* = 15 [Transgenic (Tg) with PBS] *N* = 15 (Tg with hNTSCs) *N* = 15 (Tg with hBM-MSCs)	Alizarin Red S staining; Oil Red/Safranin O staning; PET/CT imaging; IF/IHF staining; Flow cytometry; WB/ELISA; MWM trial	hNTSC application could reduce Aβ levels through modulating autophagic capacity.	Lim et al., 2021
Mir233	Animal experiments Group 1 (C57BL/6 mice) Group 2 (mir233−/− mice); *In vitro* model	Histopathology and IHC; Intracellular cytokine staining; TEM/miRNA prediction; Transient transfection; GFP-LC3 analysis; WB/RT-PCR	Mir233 regulates autophagy *via* modulating ATG16L1 expression.	Li Y. et al., 2019

Patients with AD exhibit defective autophagy and high levels of ubiquitin-binding enzyme 2c (Ube2c) in neurons (Li T. et al., 2022). In AD mouse model, microglia autophagy was significantly enhanced after knocking down Ube2c, which encodes shUbe2c in AAV2. Consistently, agomelatine (AGO) inhibited Ube2c and induced improvement of synaptic plasticity and cognitive performance (Li T. et al., 2022), suggesting that Ube2c inhibitor may provide valuable insights in AD research.

Transplantation of human neural crest-derived nasal turbinate stem cells (hNTSCs) reduced Aβ plaque deposition and Aβ levels in the brains of AD mice by enhancing autophagy, modulating inflammatory microglia status, and promoting the secretion of anti-inflammatory cytokine IL-10 (Lim et al., 2021). Meantime, hNTSCs retain multiple biological characteristics (Hwang et al., 2014) and are capable of generating various mesenchymal phenotypes *in vitro* under specific conditions (Lim et al., 2021), indicating their therapeutic potencies from aspects of neuroprotection and neuro-regeneration.

Targeting PPARA receptors was highlighted in recent NDD-related investigations. The nuclear receptor peroxisome proliferator-activated receptor α (PPARA/PPARα) is encoded by the PPARA gene (Mandard et al., 2004). PPARA is a key regulator of energy metabolism, mitochondrial function and peroxisomal function (Vamecq and Latruffe, 1999). PPARA has been also shown to be a positive regulator of cellular autophagy and mediates the increase in autophagy and autophagic flux in microglia and astrocytes after activated by the PPARA agonists gemfibrozil or Wy14643. Additionally, PPARA activation significantly reduced amyloid accumulation in AD mice’s hippocampal and cortical areas (Luo et al., 2020). Notably, amyloid plaque clearance induced by microglia and astrocyte was directly mediated by activated PPARA, indicating its potential efficacy in Aβ phagocytic uptake (Guillot-Sestier et al., 2015; Wang et al., 2015).

Several studies have suggested that manipulations of inflammation resolution (Yin et al., 2019), MEF2A enhancer methylation levels (Li H. et al., 2021), Clk1 activity regulation (Yan et al., 2017), neural TLR2/4 pathway (Chung et al., 2022), and MEJc (methanolic extract obtained from the leaves of J. curcas L.) (Bastos et al., 2021) are potential therapeutic strategies for treating aging-related NDDs, all of which were directly or indirectly correlated to the process of microglia autophagy. Further researches on these potential targets might focus on the specific mechanisms between the modulators and autophagy in nerual cells.

## 5. Conclusion

Here, we provided an overview of the relationship between microglia autophagy and NDDs. The onset and progression of NDDs are associated with the accumulation of abnormal substances in the nervous system (Cohen and Paul, 1963; Ross and Tabrizi, 2011). Recent studies revealed that microglia autophagy removes harmful substances and abnormal aggregates produced by neurons in the nervous system and acts as a neuroprotective agent (Komatsu et al., 2006; Matarin et al., 2015), which can help treat NDDs or control their progression. Meantime, manipulation of microglia autophagy also interrupts neuroinflammation in NDDs (Deretic et al., 2013; Deretic and Levine, 2018), maintain a state of equilibrium, and prevent disease progression. Therefore, the balance between microglia autophagy and neuroinflammation is of critical importance in NDDs. Noticeably, potential drugs such as Ka, melatonin and Spermidine have been shown to balance microglia autophagy and neuroinflammation in NDDs (Han et al., 2019; Chen et al., 2021; Freitag et al., 2022). However, the mechanisms of interaction between microglia autophagy and neurons have not been sufficiently elucidated, such as how microglia autophagy remove toxic substances produced by neurons or glial cells or how microglia autophagy counteract abnormal neuronal death. More in-depth studies remain to be completed in this area.

We summarized recent relevant studies and identified promising therapeutic approaches and drugs focusing on microglia autophagy. Most of them are *in vivo* or *in vitro* trials, with limited clinical trials. As the aspect of the nanodrug development in last decades, there were several teams developing nanodrugs that modulate microglia autophagy, such as and MCPZFS NPs, CS-AT NPs and PAMAM (Malviya et al., 2021; Yuan et al., 2022; Zhong et al., 2022). The Aβ recruitment possessed by MCPZFS NPs provides a new direction for future research on nanodrugs that help clear abnormal protein aggregation in NDDs. PAMAM also merits more attention with its excellent permeability (Zhong et al., 2022). However, nanomedicines have many limitations, such as the variability of their molecular structures and uncertainty in drug targeting (Liu et al., 2020). It is expected that more nanomedicines with high targeting, permeability, and recruitment ability would be focused in the future.

## Author contributions

ML, HY, and PS conceived and designed the manuscript. ML, HY, QX, and ZX contributed to the drafting and writing of the manuscript. ML and PS participated in the pictures drawing. HY and QX contributed to the creation of the tables. PS, ML, and ZX contributed to the critical revision of the manuscript for important intellectual content. All authors approved the final version of the manuscript to be published and agreed to be accountable for all aspects of the work.

## Footnotes

1
https://www.DisGeNET.org/home/

2
http://www.autophagy.lu/index.html

## References

[B1] Adams J. M. Cory S. (2001). Life-or-death decisions by the Bcl-2 protein family. *Trends Biochem. Sci.* 26 61–66. 10.1016/S0968-0004(00)01740-0 11165519

[B2] Albani D. Pupillo E. Bianchi E. Chierchia A. Martines R. Forloni G. (2016). The role of single-nucleotide variants of the energy metabolism-linked genes Sirt3. Ppargc1A and Apoe in amyotrophic lateral sclerosis risk. *Genes Gen. Syst.* 91 301–309. 10.1266/ggs.16-00023 28239025

[B3] Alcocer-Gomez E. Casas-Barquero N. Williams M. R. Romero-Guillena S. L. Canadas-Lozano D. Bullon P. (2017). Antidepressants induce autophagy dependent-Nlrp3-inflammasome inhibition in Major depressive disorder. *Pharmacol. Res.* 121 114–121. 10.1016/j.phrs.2017.04.028 28465217

[B4] Anand P. Singh B. (2013). A review on cholinesterase inhibitors for Alzheimer’s disease. *Arch. Pharm. Res.* 36 375–399. 10.1007/s12272-013-0036-3 [Epub ahead of print]. 23435942

[B5] Andres-Alonso M. Kreutz M. R. Karpova A. (2021). Autophagy and the endolysosomal system in presynaptic function. *Cell. Mol. Life Sci.* 78 2621–2639. 10.1007/s00018-020-03722-5 33340068 PMC8004491

[B6] Bai Y. J. Dergham P. Nedev H. Xu J. Galan A. Rivera J. C. (2010). Chronic and acute models of retinal neurodegeneration trka activity are neuroprotective whereas p75(ntr) activity is neurotoxic through a paracrine mechanism. *J. Biol. Chem.* 285 39392–39400. 10.1074/jbc.M110.147801 20943663 PMC2998128

[B7] Bastos E. M. S. Silva A. B. Coelho P. L. C. Borges J. M. P. Silva V. D. A. Cunha V. H. M. (2021). Anti-inflammatory activity of Jatropha curcas L. in brain glial cells primary cultures. *J. Ethnopharmacol.* 264:12. 10.1016/j.jep.2020.113201 32814081

[B8] Bellezza I. Giambanco I. Minelli A. Donato R. (2018). Nrf2-Keap1 signaling in oxidative and reductive stress. *Biochim. Et Biophys. Acta Mol. Cell Res.* 1865 721–733. 10.1016/j.bbamcr.2018.02.010 29499228

[B9] Borgonetti V. Benatti C. Governa P. Isoldi G. Pellati F. Alboni S. (2022). Non-psychotropic cannabis sativa L. phytocomplex modulates microglial inflammatory response through Cb2 receptors-, endocannabinoids-, and Nf-kappa B-mediated signaling. *Phytother. Res.* 36 2246–2263. 10.1002/ptr.7458 35393641 PMC9325551

[B10] Boyle P. A. Yu L. Wilson R. S. Leurgans S. E. Schneider J. A. Bennett D. A. (2018). Person-specific contribution of neuropathologies to cognitive loss in old age. *Ann. Neurol.* 83 74–83. 10.1002/ana.25123 29244218 PMC5876116

[B11] Brodaty H. Breteler M. M. B. Dekosky S. T. Dorenlot P. Fratiglioni L. Hock C. (2011). The world of dementia beyond 2020. *J. Am. Geriat. Soc.* 59 923–927. 10.1111/j.1532-5415.2011.03365.x 21488846

[B12] Broz P. Dixit V. M. (2016). Inflammasomes: Mechanism of assembly, regulation and signalling. *Nat. Rev. Immunol.* 16 407–420. 10.1038/nri.2016.58 27291964

[B13] Burrell J. R. Halliday G. M. Kril J. J. Ittner L. M. Gotz J. Kiernan M. C. (2016). The frontotemporal dementia-motor neuron disease continuum. *Lancet* 388 919–931. 10.1016/S0140-6736(16)00737-6 26987909

[B14] Cerminara C. El-Malhany N. Roberto D. Lo C. A. Curatolo P. (2013). Seizures induced by desloratadine. *Second Gen Antihistamine* 44 222–224. 10.1055/s-0033-1333871 23456992

[B15] Chagas L. D. Sandre P. C. Ribeiro N. Marcondes H. Silva P. O. Savino W. (2020). Environmental signals on microglial function during brain development. Neuroplasticity, and disease. *Int. J. Mol. Sci.* 21:20. 10.3390/ijms21062111 32204421 PMC7139373

[B16] Charmpilas N. Fang E. F. Palikaras K. (2022). Mitophagy and neuroinflammation: A compelling interplay. *Curr. Neuropharmacol.* ^**^. 10.2174/1570159X20666220628153632 35762540 PMC10472808

[B17] Chen C. Y. Yang C. Wang J. Huang X. Yu H. T. Li S. M. (2021). Melatonin ameliorates cognitive deficits through improving mitophagy in a mouse model of Alzheimer’s disease. *J. Pineal Res.* 71:17. 10.1111/jpi.12774 34617321

[B18] Cheng J. B. Liao Y. J. Dong Y. Hu H. Yang N. N. Kong X. X. (2020). Microglial autophagy defect causes parkinson disease-like symptoms by accelerating inflammasome activation in mice. *Autophagy* 16 2193–2205. 10.1080/15548627.2020.1719723 32003282 PMC7751565

[B19] Chong C. M. Ke M. J. Tan Y. Huang Z. J. Zhang K. Ai N. N. (2018). Presenilin 1 deficiency suppresses autophagy in human neural stem cells through reducing gamma-secretase-independent Erk/Creb signaling. *Cell Death Disease* 9:13. 10.1038/s41419-018-0945-7 30158533 PMC6115391

[B20] Chung L. Y. R. Lin Y. T. Liu C. Tai Y. C. Lin H. Y. Lin C. H. (2022). Neuroinflammation upregulated neuronal toll-like receptors 2 and 4 to drive synucleinopathy in neurodegeneration. *Front. Pharmacol.* 13:14. 10.3389/fphar.2022.845930 35401198 PMC8987529

[B21] Codogno P. Mehrpour M. Proikas-Cezanne T. (2012). Canonical and non-canonical autophagy: Variations on a common theme of self-eating? *Nat. Rev. Mol. Cell Biol.* 13 7–12. 10.1038/nrm3249 22166994

[B22] Cohen A. S. Paul W. E. (1963). Relationship of gamma-globulin to the fibrils of secondary human amyloid. *Nature* 197 193–194. 10.1038/197193a0 14022005

[B23] Colonna M. Butovsky O. (2017). “Microglia function in the central nervous system during health and neurodegeneration,” in *Annual review of immunology*, Vol. 35 eds Littman D. R. Yokoyama W. M. (Palo Alto: Annual Reviews). 10.1146/annurev-immunol-051116-052358 PMC8167938 28226226

[B24] Condon K. J. Sabatini D. M. (2019). Nutrient regulation of mtorc1 at a glance. *J. Cell Sci.* 132:6. 10.1242/jcs.222570 31722960 PMC6857595

[B25] Crisan T. O. Plantinga T. S. van de Veerdonk F. L. Farcas M. F. Stoffels M. Kullberg B.-J. (2011). Inflammasome-independent modulation of cytokine response by autophagy in human cells. *PloS One* 6:e18666. 10.1371/journal.pone.0018666 21490934 PMC3072416

[B26] Del Rey N. L. G. Quiroga-Varela A. Garbayo E. Carballo-Carbajal I. Fernandez-Santiago R. Monje M. H. G. (2018). Advances in Parkinson’s disease: 200 years later. *Front. Neuroanat.* 12:14. 10.3389/fnana.2018.00113 30618654 PMC6306622

[B27] Deretic V. Levine B. (2018). Autophagy balances inflammation in innate immunity. *Autophagy* 14 243–251. 10.1080/15548627.2017.1402992 29165043 PMC5902214

[B28] Deretic V. Saitoh T. Akira S. (2013). Autophagy in infection, inflammation and immunity. *Nat. Rev. Immunol.* 13 722–737. 10.1038/nri3532 24064518 PMC5340150

[B29] Ding H. G. Li Y. Chen S. L. Wen Y. Zhang S. Y. Luo E. S. (2022). Fisetin ameliorates cognitive impairment by activating mitophagy and suppressing neuroinflammation in rats with sepsis-associated encephalopathy. *Cns Neurosci. Ther.* 28 247–258. 10.1111/cns.13765 34837343 PMC8739041

[B30] Duan Z. X. Shi Y. Lin Q. Hamai A. Mehrpour M. Gong C. (2022). Autophagy-associated immunogenic modulation and its applications in cancer therapy. *Cells* 11:15. 10.3390/cells11152324 35954167 PMC9367255

[B31] Dupont N. Nascimbeni A. C. Morel E. Codogno P. (2017). “Molecular mechanisms of noncanonical autophagy,” in *International review of cell and molecular biology*, Vol. 328 ed. Galluzzi L. (San Diego: Elsevier Academic Press Inc). 10.1016/bs.ircmb.2016.08.001 28069131

[B32] Dutta K. Patel P. Julien J. P. (2018). Protective effects of Withania somnifera extract in Sod1(G93A) mouse model of amyotrophic lateral sclerosis. *Exp. Neurol.* 309 193–204. 10.1016/j.expneurol.2018.08.008 30134145

[B33] Dzamko N. Gysbers A. Perera G. Bahar A. Shankar A. Gao J. Q. (2017). Toll-like receptor 2 is increased in neurons in Parkinson’s disease brain and may contribute to alpha-synuclein pathology. *Acta Neuropathol.* 133 303–319. 10.1007/s00401-016-1648-8 27888296 PMC5250664

[B34] El-Khider F. McDonald C. (2016). Links of autophagy dysfunction to inflammatory bowel disease onset. *Digest. Diseas.* 34 27–34. 10.1159/000442921 26982478 PMC5378153

[B35] Eshraghi M. Adlimoghaddam A. Mahmoodzadeh A. Sharifzad F. Yasavoli-Sharahi H. Lorzadeh S. (2021). Alzheimer’s disease pathogenesis: Role of autophagy and mitophagy focusing in microglia. *Int. J. Mol. Sci.* 22:36. 10.3390/ijms22073330 33805142 PMC8036323

[B36] Espay A. J. Morgante F. Merola A. Fasano A. Marsili L. Fox S. H. (2018). Levodopa-induced dyskinesia in Parkinson disease: Current and evolving concepts. *Ann. Neurol.* 84 797–811. 10.1002/ana.25364 30357892

[B37] Fabrizi C. Pompili E. Somma F. De V. S. Ciraci V. Artico M. (2017). Lithium limits trimethyltin-induced cytotoxicity and proinflammatory response in microglia without affecting the concurrent autophagy impairment. *J. Appl. Toxicol.* 37 207–213. 10.1002/jat.3344 27226005

[B38] Forno L. S. (1996). Neuropathology of Parkinson’s disease. *J. Neuropathol. Exp. Neurol.* 55 259–272. 10.1097/00005072-199603000-00001 8786384

[B39] Franco R. Fernandez-Suarez D. (2015). Alternatively activated microglia and macrophages in the central nervous system. *Prog. Neurobiol.* 131 65–86. 10.1016/j.pneurobio.2015.05.003 26067058

[B40] Frank-Cannon T. C. Alto L. T. Mcalpine F. E. Tansey M. G. (2009). Does neuroinflammation fan the flame in neurodegenerative diseases? *Mol. Neurodegener.* 4:13. 10.1186/1750-1326-4-47 19917131 PMC2784760

[B41] Freitag K. Sterczyk N. Wendlinger S. Obermayer B. Schulz J. Farztdinov V. (2022). Spermidine reduces neuroinflammation and soluble amyloid beta in an Alzheimer’s disease mouse model. *J. Neuroinflammation* 19:19. 10.1186/s12974-022-02534-7 35780157 PMC9250727

[B42] Frigerio R. Fujishiro H. Ahn T. B. Josephs K. A. Maraganore D. M. Delledonne A. (2011). Incidental Lewy body disease: Do some cases represent a preclinical stage of dementia with Lewy bodies? *Neurobiol. Aging* 32 857–863. 10.1016/j.neurobiolaging.2009.05.019 19560232 PMC3366193

[B43] Ganesan D. Cai Q. (2021). Understanding amphisomes. *Biochem. J.* 478 1959–1976. 10.1042/BCJ20200917 34047789 PMC8935502

[B44] Ganie S. A. Dar T. A. Bhat A. H. Dar K. B. Anees S. Zargar M. A. (2016). Melatonin: A potential anti-oxidant therapeutic agent for mitochondrial dysfunctions and related disorders. *Rejuven. Res.* 19 21–40. 10.1089/rej.2015.1704 26087000

[B45] Ganley I. G. Lam D. H. Wang J. R. Ding X. J. Chen S. Jiang X. J. (2009). Ulk1 center dot Atg13 center dot Fip200 complex mediates mtor signaling and is essential for autophagy. *J. Biol. Chem.* 284 12297–12305. 10.1074/jbc.M900573200 19258318 PMC2673298

[B46] Ge X. Wang Y. Yu S. Cao X. Chen Y. Cheng Q. (2021). Anti-inflammatory activity of a polypeptide fraction from achyranthes bidentate in amyloid beta oligomers induced model of Alzheimer’s disease. *Front. Pharmacol.* 12:716177. 10.3389/fphar.2021.716177 34456729 PMC8397449

[B47] Ghanbarpour A. Valverde D. P. Melia T. J. Reinisch K. M. (2021). A model for a partnership of lipid transfer proteins and scramblases in membrane expansion and organelle biogenesis. *Proc. Natl. Acad. Sci. U.S.A.* 118:3. 10.1073/pnas.2101562118 33850023 PMC8072408

[B48] Giorgi C. Marchi S. Simoes I. C. M. Ren Z. Y. Morciano G. Perrone M. (2018). “Mitochondria and reactive oxygen species in aging and age-related diseases,” in *Mitochondria and longevity*, eds Lopezotin C. Galluzzi L. (San Diego: Elsevier Academic Press Inc). 10.1016/bs.ircmb.2018.05.006

[B49] Glick D. Barth S. Macleod K. F. (2010). Autophagy: Cellular and molecular mechanisms. *J. Pathol.* 221 3–12. 10.1002/path.2697 20225336 PMC2990190

[B50] Gu H. F. Nie Y. X. Tong Q. Z. Tang Y. L. Zeng Y. Jing K. Q. (2014). Epigallocatechin-3-Gallate attenuates impairment of learning and memory in chronic unpredictable mild stress-treated rats by restoring hippocampal autophagic flux. *Plos One* 9:e112683. 10.1371/journal.pone.0112683 25393306 PMC4231069

[B51] Guillot-Sestier M. V. Doty K. R. Gate D. Rodriguez J. Leung B. P. Rezai-Zadeh K. (2015). Il10 deficiency rebalances innate immunity to mitigate Alzheimer-like pathology. *Neuron* 85 534–548. 10.1016/j.neuron.2014.12.068 25619654 PMC4352138

[B52] Hahm E. R. Singh S. V. (2013). Autophagy fails to alter withaferin a-mediated lethality in human breast cancer cells. *Curr. Cancer Drug Target.* 13 640–650. 10.2174/15680096113139990039 23607597 PMC3723758

[B53] Han X. J. Sun S. F. Sun Y. M. Song Q. Q. Zhu J. L. Song N. S. (2019). Small molecule-driven Nlrp3 inflammation inhibition via interplay between ubiquitination and autophagy: Implications for Parkinson disease. *Autophagy* 15 1860–1881. 10.1080/15548627.2019.1596481 30966861 PMC6844502

[B54] Hara T. Nakamura K. Matsui M. Yamamoto A. Nakahara Y. Suzuki-Migishima R. (2006). Suppression of basal autophagy in neural cells causes neurodegenerative disease in mice. *Nature* 441 885–889. 10.1038/nature04724 16625204

[B55] Haukedal H. Freude K. (2019). Implications of microglia in amyotrophic lateral sclerosis and frontotemporal dementia. *J. Mol. Biol.* 431 1818–1829. 10.1016/j.jmb.2019.02.004 30763568

[B56] He C. C. Klionsky D. J. (2009). Regulation mechanisms and signaling pathways of autophagy. *Annu. Rev. Genet.* 43 67–93. 10.1146/annurev-genet-102808-114910 19653858 PMC2831538

[B57] He D. W. Hu G. Q. Zhou A. Liu Y. Huang B. X. Su Y. C. (2022). Echinocystic acid inhibits inflammation and exerts neuroprotective effects in mptp-induced Parkinson’s disease model mice. *Front. Pharmacol.* 12:787771. 10.3389/fphar.2021.787771 35126128 PMC8807489

[B58] Heckmann B. L. Green D. R. (2019). Lc3-associated phagocytosis at a glance. *J. Cell Sci.* 132:6. 10.1242/jcs.222984 30787029 PMC6432721

[B59] Heckmann B. L. Teubner B. J. W. Boada-Romero E. Tummers B. Guy C. Fitzgerald P. (2020). Noncanonical function of an autophagy protein prevents spontaneous Alzheimer’s disease. *Sci. Adv.* 6:12. 10.1126/sciadv.abb9036 32851186 PMC7428329

[B60] Heckmann B. L. Teubner B. J. W. Tummers B. Boada-Romero E. Harris L. Yang M. (2019). Lc3-associated endocytosis facilitates beta-amyloid clearance and mitigates neurodegeneration in murine Alzheimer’s disease. *Cell* 178 536–551. 10.1016/j.cell.2019.05.056 31257024 PMC6689199

[B61] Heneka M. T. (2017). Inflammasome activation and innate immunity in Alzheimer’s disease. *Brain Pathol.* 27 220–222. 10.1111/bpa.12483 28019679 PMC8029274

[B62] Hindle J. V. (2010). Ageing, neurodegeneration and Parkinson’s disease. *Age Ageing* 39 156–161. 10.1093/ageing/afp223 20051606

[B63] Holbrook J. A. Jarosz-Griffiths H. H. Caseley E. Lara-Reyna S. Poulter J. A. Williams-Gray C. H. (2021). Neurodegenerative Disease and the Nlrp3 Inflammasome. *Front. Pharmacol.* 12:15. 10.3389/fphar.2021.643254 33776778 PMC7987926

[B64] Holczer M. Besze B. Zambo V. Csala M. Banhegyi G. Kapuy O. (2018). Epigallocatechin-3-Gallate (Egcg) Promotes autophagy-dependent survival via influencing the balance of mtor-ampk pathways upon endoplasmic reticulum stress. *Oxid. Med. Cell. Long.* 2018:15. 10.1155/2018/6721530 29636854 PMC5831959

[B65] Hosokawa N. Hara T. Kaizuka T. Kishi C. Takamura A. Miura Y. (2009). Nutrient-dependent mtorc1 Association with the Ulk1-Atg13-Fip200 Complex Required for Autophagy. *Mol. Biol. Cell* 20 1981–1991. 10.1091/mbc.e08-12-1248 19211835 PMC2663915

[B66] Houtman J. Freitag K. Gimber N. Schmoranzer J. Heppner F. L. Jendrach M. (2019). Beclin1-driven autophagy modulates the inflammatory response of microglia via Nlrp3. *Embo J.* 38:15. 10.15252/embj.201899430 30617086 PMC6376276

[B67] Hwang S. H. Park S. H. Choi J. Lee D. C. Oh J. H. Kim S. W. (2014). Characteristics of mesenchymal stem cells originating from the bilateral inferior turbinate in humans with nasal septal deviation. *Plos One* 9:e100219. 10.1371/journal.pone.0100219 24926874 PMC4057459

[B68] Inoki K. Zhu T. Guan K.-L. (2003). Tsc2 mediates cellular energy response to control cell growth and survival. *Cell* 115 577–590. 10.1016/S0092-8674(03)00929-2 14651849

[B69] Itakura E. Kishi C. Inoue K. Mizushima N. (2008). Beclin 1 forms two distinct phosphatidylinositol 3-kinase complexes with mammalian Atg14 and Uvrag. *Mol. Biol. Cell* 19 5360–5372. 10.1091/mbc.e08-01-0080 18843052 PMC2592660

[B70] Jaiswal M. K. (2019). Riluzole and edaravone: A tale of two amyotrophic lateral sclerosis drugs. *Med. Res. Rev.* 39 733–748. 10.1002/med.21528 30101496

[B71] Javidnia M. Hebron M. L. Xin Y. Kinney N. G. Moussa C. E. H. (2017). Pazopanib reduces phosphorylated tau levels and alters astrocytes in a mouse model of tauopathy. *J. Alzheimers Disease* 60 461–481. 10.3233/JAD-170429 28869476 PMC5757517

[B72] Jin M. H. Shiwaku H. Tanaka H. Obita T. Ohuchi S. Yoshioka Y. (2021). Tau activates microglia via the Pqbp1-cgas-Sting pathway to promote brain inflammation. *Nat. Commun.* 12:22. 10.1038/s41467-021-26851-2 34782623 PMC8592984

[B73] Jin M. M. Wang F. Qi D. Liu W. W. Gu C. Mao C. J. (2018). A critical role of autophagy in regulating microglia polarization in neurodegeneration. *Front. Aging Neurosci.* 10:378. 10.3389/fnagi.2018.00378 30515090 PMC6256089

[B74] Johnson M. E. Bobrovskaya L. (2015). An update on the rotenone models of Parkinson’s disease: Their ability to reproduce the features of clinical disease and model gene-environment interactions. *Neurotoxicology* 46 101–116. 10.1016/j.neuro.2014.12.002 25514659

[B75] Joshi T. Kumar V. Kaznacheyeva E. V. Jana N. R. (2021). Withaferin a induces heat shock response and ameliorates disease progression in a mouse model of Huntington’s disease. *Mol. Neurobiol.* 58 3992–4006. 10.1007/s12035-021-02397-8 33904021

[B76] Katsnelson A. De S. Zoghbi H. Y. (2016). Neurodegeneration: From cellular concepts to clinical applications. *Sci. Transl. Med.* 8:5. 10.1126/scitranslmed.aal2074 27831899

[B77] Keren-Shaul H. Spinrad A. Weiner A. Matcovitch-Natan O. Dvir-Szternfeld R. Ulland T. K. (2017). A unique microglia type associated with restricting development of Alzheimer’s disease. *Cell* 169 1276.e–1290.e. 10.1016/j.cell.2017.05.018 28602351

[B78] Kim J. Kundu M. Viollet B. Guan K. L. (2011). Ampk and mtor regulate autophagy through direct phosphorylation of Ulk1. *Nat. Cell Biol.* 13 132–U71. 10.1038/ncb2152 21258367 PMC3987946

[B79] Klein C. Westenberger A. (2012). Genetics of Parkinson’s disease. *Cold Spring Harb. Pers. Med.* 2:15. 10.1101/cshperspect.a008888 22315721 PMC3253033

[B80] Koch Y. Helferich A. M. Steinacker P. Oeckl P. Walther P. Weishaupt J. H. (2016). Aggregated alpha-Synuclein Increases S0D1 Oligomerization in a Mouse Model of Amyotrophic Lateral Sclerosis. *Am. J. Pathol.* 186 2152–2161. 10.1016/j.ajpath.2016.04.008 27322773

[B81] Kodali M. Attaluri S. Madhu L. N. Shuai B. Upadhya R. Gonzalez J. J. (2021). Metformin treatment in late middle age improves cognitive function with alleviation of microglial activation and enhancement of autophagy in the hippocampus. *Aging Cell* 20:19. 10.1111/acel.13277 33443781 PMC7884047

[B82] Komatsu M. Waguri S. Chiba T. Murata S. Iwata J. Tanida I. (2006). Loss of autophagy in the central nervous system causes neurodegeneration in mice. *Nature* 441 880–884. 10.1038/nature04723 16625205

[B83] Kuijpers M. Tehran D. A. Haucke V. Soykan T. (2021). The axonal endolysosomal and autophagic systems. *J. Neurochem.* 158 589–602. 10.1111/jnc.15287 33372296

[B84] Kuusisto E. Suuronen T. Salminen A. (2001). Ubiquitin-binding protein p62 expression is induced during apoptosis and proteasomal inhibition in neuronal cells. *Biochem. Biophys. Res. Commun.* 280 223–228. 10.1006/bbrc.2000.4107 11162503

[B85] Lautrup S. Lou G. F. Aman Y. Nilsen H. Tao J. Fang E. F. (2019). Microglial mitophagy mitigates neuroinflammation in Alzheimer’s disease. *Neurochem. Int.* 129:4. 10.1016/j.neuint.2019.104469 31100304

[B86] Lee Y. K. Lee J. A. (2016). Role of the mammalian Atg8/Lc3 family in autophagy: Differential and compensatory roles in the spatiotemporal regulation of autophagy. *BMB Rep.* 49 424–430. 10.5483/BMBRep.2016.49.8.081 27418283 PMC5070729

[B87] Lemasters J. J. (2014). Variants of mitochondrial autophagy: Types 1 and 2 mitophagy and micromitophagy (Type 3). *Redox Biol.* 2 749–754. 10.1016/j.redox.2014.06.004 25009776 PMC4085350

[B88] Lemasters J. J. Zhong Z. (2018). Mitophagy in hepatocytes: Types, initiators and role in adaptive ethanol metabolism. *Liver Res.* 2 125–132. 10.1016/j.livres.2018.09.005 31157120 PMC6541449

[B89] Lerner C. A. Sundar I. K. Rahman I. (2016). Mitochondrial redox system, dynamics, and dysfunction in lung inflammaging and Copd. *Int. J. Biochem. Cell Biol.* 81 294–306. 10.1016/j.biocel.2016.07.026 27474491 PMC5154857

[B90] Li D. D. Zheng C. Q. Zhang F. Shi J. S. (2022). Potential neuroprotection by Dendrobium nobile Lindl alkaloid in Alzheimer’s disease models. *Neural Regener. Res.* 17 972–977. 10.4103/1673-5374.324824 34558510 PMC8552836

[B91] Li T. Su Q. Zhang Z. A. Zhang Y. L. Yang M. X. Wang Z. R. (2022). Ube2c-inhibition alleviated amyloid pathology and memory deficits in App/Ps1 mice model of Ad. *Prog. Neurobiol.* 215:12. 10.1016/j.pneurobio.2022.102298 35671859

[B92] Li H. Wang F. Guo X. Q. Jiang Y. G. (2021). Decreased Mef2A expression regulated by its enhancer methylation inhibits autophagy and may play an important role in the progression of Alzheimer’s disease. *Front. Neurosci.* 15:682247. 10.3389/fnins.2021.682247 34220439 PMC8242211

[B93] Li S. H. Colson T. L. L. Abd-Elrahman K. S. Ferguson S. S. G. (2021). Metabotropic glutamate receptor 2/3 activation improves motor performance and reduces pathology in heterozygous zQ175 huntington disease mice. *J. Pharmacol. Exp. Ther.* 379 74–84. 10.1124/jpet.121.000735 34330748

[B94] Li J. W. Zong Y. Cao X. P. Tan L. Tan L. (2018). Microglial priming in Alzheimer’s disease. *Ann. Transl. Med.* 6:14. 10.21037/atm.2018.04.22 29951498 PMC5994530

[B95] Li W. H. Li Y. J. Siraj S. Jin H. J. Fan Y. Y. Yang X. R. (2019). Fun14 domain-containing 1-mediated mitophagy suppresses hepatocarcinogenesis by inhibition of inflammasome activation in mice. *Hepatology* 69 604–621. 10.1002/hep.30191 30053328

[B96] Li Y. Zhou D. M. Ren Y. H. Zhang Z. M. Guo X. D. Ma M. K. (2019). Mir223 restrains autophagy and promotes Cns inflammation by targeting Atg16L1. *Autophagy* 15 478–492. 10.1080/15548627.2018.1522467 30208760 PMC6351131

[B97] Li W. W. Li J. Bao J. K. (2012). Microautophagy: Lesser-known self-eating. *Cell. Mol. Life Sci.* 69 1125–1136. 10.1007/s00018-011-0865-5 22080117 PMC11114512

[B98] Li X. L. Li K. Chu F. X. Huang J. Yang Z. (2020). Graphene oxide enhances beta-amyloid clearance by inducing autophagy of microglia and neurons. *Chem. Biol. Inter.* 325:10. 10.1016/j.cbi.2020.109126 32430275

[B99] Liang J. Y. Shao S. H. Xu Z. X. Hennessy B. Ding Z. Y. Larrea M. (2007). The energy sensing Lkb1-Ampk pathway regulates p27(kip1) phosphorylation mediating the decision to enter autophagy or apoptosis. *Nat. Cell Biol.* 9 218–U125. 10.1038/ncb1537 17237771

[B100] Lim J. Y. Park S. I. Park S. A. Jeon J. H. Jung H. Y. Yon J. M. (2021). Potential application of human neural crest-derived nasal turbinate stem cells for the treatment of neuropathology and impaired cognition in models of Alzheimer’s disease. *Stem Cell Res. Ther.* 12:18. 10.1186/s13287-021-02489-1 34256823 PMC8278635

[B101] Liu R. Yang J. Liu L. Lu Z. Shi Z. Ji W. (2020). An “amyloid-β cleaner” for the treatment of alzheimer’s disease by normalizing microglial dysfunction. *Adv. Sci.* 7:1901555. 10.1002/advs.201901555 31993283 PMC6974948

[B102] Lorente Pons A. Higginbottom A. Cooper-Knock J. Alrafiah A. Alofi E. Kirby J. (2020). Oligodendrocyte pathology exceeds axonal pathology in white matter in human amyotrophic lateral sclerosis. *J. Pathol.* 251 262–271. 10.1002/path.5455 32391572

[B103] Lu J. Wang C. Cheng X. Wang R. Yan X. He P. (2022). A breakdown in microglial metabolic reprogramming causes internalization dysfunction of alpha-synuclein in a mouse model of Parkinson’s disease. *J. Neuroinflammation* 19:113. 10.1186/s12974-022-02484-0 35599331 PMC9124408

[B104] Lu J. Zhang C. Z. Lv J. L. Zhu X. L. Jiang X. W. Lu W. Q. (2021). Antiallergic drug desloratadine as a selective antagonist of 5ht(2A) receptor ameliorates pathology of Alzheimer’s disease model mice by improving microglial dysfunction. *Aging Cell* 20:17. 10.1111/acel.13286 33369003 PMC7811850

[B105] Lu M. Su C. J. Qiao C. Bian Y. Q. Ding J. H. Hu G. (2016). Metformin prevents dopaminergic neuron death in Mptp/P-Induced mouse model of Parkinson’s disease via autophagy and mitochondrial ros clearance. *Int. J. Neuropsychopharmacol.* 19:11. 10.1093/ijnp/pyw047 27207919 PMC5043649

[B106] Luengo E. Buendia I. Fernandez-Mendivil C. Trigo-Alonso P. Negredo P. Michalska P. (2019). Pharmacological doses of melatonin impede cognitive decline in tau-related Alzheimer models, once tauopathy is initiated, by restoring the autophagic flux. *J. Pineal Res.* 67:16. 10.1111/jpi.12578 30943316

[B107] Luo R. C. Su L. Y. Li G. Y. Yang J. Liu Q. J. Yang L. X. (2020). Activation of Ppara-mediated autophagy reduces Alzheimer disease-like pathology and cognitive decline in a murine model. *Autophagy* 16 52–69. 10.1080/15548627.2019.1596488 30898012 PMC6984507

[B108] Lv L. L. Liu J. Li L. S. Jin F. Xu Y. Y. Wu Q. (2020). Dendrobium nobile lindl. alkaloids ameliorate cognitive dysfunction in senescence accelerated Samp8 mice by decreasing amyloid-beta aggregation and enhancing autophagy activity. *J. Alzheimers Dis.* 76 657–669. 10.3233/JAD-200308 32538851

[B109] Lv X. P. Li W. Luo Y. Wang D. D. Zhu C. Q. Huang Z. X. (2013). Exploring the differences between mouse mA beta(1-42) and human hA beta(1-42) for Alzheimer’s disease related properties and neuronal cytotoxicity. *Chem. Commun.* 49 5865–5867. 10.1039/c3cc40779a 23700581

[B110] Lynch-Day M. A. Klionsky D. J. (2010). The Cvt pathway as a model for selective autophagy. *FEBS Lett.* 584 1359–1366. 10.1016/j.febslet.2010.02.013 20146925 PMC2843786

[B111] Malviya R. Fuloria S. Verma S. Subramaniyan V. Sathasivam K. V. Kumarasamy V. (2021). Commercial utilities and future perspective of nanomedicines. *Peerj* 9:30. 10.7717/peerj.12392 34820175 PMC8607930

[B112] Mandard S. Muller M. Kersten S. (2004). Peroxisome proliferator-activated receptor alpha target genes. *Cell. Mol. Life Sci.* 61 393–416. 10.1007/s00018-003-3216-3 14999402 PMC11138883

[B113] Martinez J. Almendinger J. Oberst A. Ness R. Dillon C. P. Fitzgerald P. (2011). Microtubule-associated protein 1 light chain 3 alpha (Lc3)-associated phagocytosis is required for the efficient clearance of dead cells. *Proc. Natl. Acad. Sci. U.S.A.* 108 17396–17401. 10.1073/pnas.1113421108 21969579 PMC3198353

[B114] Martinez J. Malireddi R. K. S. Lu Q. Cunha L. D. Pelletier S. Gingras S. (2015). Molecular characterization of Lc3-associated phagocytosis reveals distinct roles for Rubicon. Nox2 and autophagy proteins. *Nat. Cell Biol.* 17 893–906. 10.1038/ncb3192 26098576 PMC4612372

[B115] Martinez-Martin P. Rodriguez-Blazquez C. Forjaz M. J. (2012). Quality of life and burden in caregivers for patients with Parkinson’s disease: Concepts, assessment and related factors. *Expert Rev. Pharmacoecon. Outcomes Res.* 12 221–230. 10.1586/erp.11.106 22458623

[B116] Martinez-Vicente M. Talloczy Z. Wong E. Tang G. M. Koga H. Kaushik S. (2010). Cargo recognition failure is responsible for inefficient autophagy in Huntington’s disease. *Nat. Neurosci.* 13 567–U74. 10.1038/nn.2528 20383138 PMC2860687

[B117] Matarin M. Salih D. A. Yasvoina M. Cummings D. M. Guelfi S. Liu W. F. (2015). A genome-wide gene-expression analysis and database in transgenic mice during development of amyloid or tau pathology. *Cell Rep.* 10 633–644. 10.1016/j.celrep.2014.12.041 25620700

[B118] McCauley M. E. O’rourke J. G. Yanez A. Markman J. L. Ho R. Wang X. C. (2020). C9orf72 in myeloid cells suppresses Sting-induced inflammation. *Nature* 585 96–101. 10.1038/s41586-020-2625-x 32814898 PMC7484469

[B119] McLelland G. L. Soubannier V. Chen C. X. Mcbride H. M. Fon E. A. (2014). Parkin and Pink1 function in a vesicular trafficking pathway regulating mitochondrial quality control. *EMBO J.* 33 282–295. 10.1002/embj.201385902 24446486 PMC3989637

[B120] Moscariello P. Ng D. Y. W. Jansen M. Weil T. Luhmann H. J. Hedrich J. (2018). Brain delivery of multifunctional dendrimer protein bioconjugates. *Adv. Sci. (Weinh)* 5:1700897. 10.1002/advs.201700897 29876217 PMC5979778

[B121] Mullock B. M. Perez J. H. Kuwana T. Gray S. R. Luzio J. P. (1994). Lysosomes can fuse with a late endosomal compartment in a cell-free system from rat liver. *J. Cell Biol.* 126 1173–1182. 10.1083/jcb.126.5.1173 7520447 PMC2120167

[B122] Munz C. (2016). Autophagy proteins in antigen processing for presentation on Mhc molecules. *Immunol. Rev.* 272 17–27. 10.1111/imr.12422 27319339

[B123] Munz C. (2018). Non-canonical functions of macroautophagy proteins during endocytosis by myeloid antigen presenting cells. *Front. Immunol.* 9:2765. 10.3389/fimmu.2018.02765 30542350 PMC6277852

[B124] Nakatogawa H. (2020). Mechanisms governing autophagosome biogenesis. *Nat. Rev. Mol. Cell Biol.* 21 439–458. 10.1038/s41580-020-0241-0 32372019

[B125] Nelson P. T. Head E. Schmitt F. A. Davis P. R. Neltner J. H. Jicha G. A. (2011). Alzheimer’s disease is not “brain aging”: Neuropathological, genetic, and epidemiological human studies. *Acta Neuropathol.* 121 571–587. 10.1007/s00401-011-0826-y 21516511 PMC3179861

[B126] Niu H. Alvarez-Alvarez I. Guillen-Grima F. Aguinaga-Ontoso I. (2017). Prevalence and incidence of Alzheimer’s disease in Europe: A meta -analysis. *Neurologia* 32 523–532. 10.1016/j.nrl.2016.02.016 27130306

[B127] Nixon R. A. Yang D. S. (2011). Autophagy failure in Alzheimer’s disease-locating the primary defect. *Neurobiol. Dis.* 43 38–45. 10.1016/j.nbd.2011.01.021 21296668 PMC3096679

[B128] O’Brien W. T. Klein P. S. (2009). Validating Gsk3 as an in vivo target of lithium action. *Biochem. Soc. Trans.* 37 1133–1138. 10.1042/BST0371133 19754466 PMC2747042

[B129] Ochaba J. Lukacsovich T. Csikos G. Zheng S. Q. Margulis J. Salazar L. (2014). Potential function for the Huntingtin protein as a scaffold for selective autophagy. *Proc. Natl. Acad. Sci. U.S.A.* 111 16889–16894. 10.1073/pnas.1420103111 25385587 PMC4250109

[B130] Pagano G. Niccolini F. Politis M. (2016). Current status of pet imaging in Huntington’s disease. *Eur. J. Nucl. Med. Mol. Imaging* 43 1171–1182. 10.1007/s00259-016-3324-6 26899245 PMC4844650

[B131] Pandi-Perumal S. R. Bahammam A. S. Brown G. M. Spence D. W. Bharti V. K. Kaur C. (2013). Melatonin antioxidative defense: Therapeutical implications for aging and neurodegenerative processes. *Neurotox. Res.* 23 267–300. 10.1007/s12640-012-9337-4 22739839

[B132] Park S. H. Lee Y. S. Yang H. J. Song G. J. (2021). Fluoxetine potentiates phagocytosis and autophagy in microglia. *Front. Pharmacol.* 12:770610. 10.3389/fphar.2021.770610 34899324 PMC8662994

[B133] Perry V. H. Nicoll J. A. R. Holmes C. (2010). Microglia in neurodegenerative disease. *Nat. Rev. Neurol.* 6 193–201. 10.1038/nrneurol.2010.17 20234358

[B134] Plaza-Zabala A. Sierra-Torre V. Sierra A. (2017). Autophagy and microglia: Novel partners in neurodegeneration and aging. *Int. J. Mol. Sci.* 18:28. 10.3390/ijms18030598 28282924 PMC5372614

[B135] Poewe W. Seppi K. Tanner C. M. Halliday G. M. Brundin P. Volkmann J. (2017). Parkinson disease. *Nat. Rev. Dis. Primers* 3:21. 10.1038/nrdp.2017.13 28332488

[B136] Proikas-Cezanne T. Ktistakis N. T. (2020). Editorial: Autophagy and ageing: Ideas. Methods, molecules. *Front. Cell Dev. Biol.* 8:2. 10.3389/fcell.2020.00141 32185175 PMC7058589

[B137] Qin Y. Qiu J. R. Wang P. Liu J. Zhao Y. Jiang F. (2021). Impaired autophagy in microglia aggravates dopaminergic neurodegeneration by regulating Nlrp3 inflammasome activation in experimental models of Parkinson’s disease. *Brain Behav. Immun.* 91 324–338. 10.1016/j.bbi.2020.10.010 33039664

[B138] Qiu J. R. Chen Y. Zhuo J. Zhang L. Liu J. Wang B. Z. (2022). Urolithin A promotes mitophagy and suppresses Nlrp3 inflammasome activation in lipopolysaccharide-induced Bv2 microglial cells and Mptp-induced Parkinson’s disease model. *Neuropharmacology* 207:15. 10.1016/j.neuropharm.2022.108963 35065082

[B139] Qiu W. Q. Ai W. Zhu F. D. Zhang Y. Guo M. S. Law B. Y. K. (2022). Polygala saponins inhibit Nlrp3 inflammasome-mediated neuroinflammation via Shp-2-Mediated mitophagy. *Free Radic. Biol. Med.* 179 76–94. 10.1016/j.freeradbiomed.2021.12.263 34933095

[B140] Rambold A. S. Lippincott-Schwartz J. (2011). Mechanisms of mitochondria and autophagy crosstalk. *Cell Cycle* 10 4032–4038. 10.4161/cc.10.23.18384 22101267 PMC3272286

[B141] Rogov V. Dotsch V. Johansen T. Kirkin V. (2014). Interactions between autophagy receptors and ubiquitin-like proteins form the molecular basis for selective autophagy. *Mol. Cell* 53 167–178. 10.1016/j.molcel.2013.12.014 24462201

[B142] Ross C. A. Tabrizi S. J. (2011). Huntington’s disease: From molecular pathogenesis to clinical treatment. *Lancet Neurol.* 10 83–98. 10.1016/S1474-4422(10)70245-3 21163446

[B143] Sahoo S. K. Parveen S. Panda J. J. (2007). The present and future of nanotechnology in human health care. *Nanomed. Nanotechnol. Biol. Med.* 3 20–31. 10.1016/j.nano.2006.11.008 17379166

[B144] Samie M. Wang X. Zhang X. L. Goschka A. Li X. R. Cheng X. P. (2013). A Trp channel in the lysosome regulates large particle phagocytosis via focal exocytosis. *Dev. Cell* 26 511–524. 10.1016/j.devcel.2013.08.003 23993788 PMC3794471

[B145] Samuvel D. J. Li L. Krishnasamy Y. Gooz M. Takemoto K. Woster P. M. (2022). Mitochondrial depolarization after acute ethanol treatment drives mitophagy in living mice. *Autophagy* 18 2671–2685. 10.1080/15548627.2022.2046457 35293288 PMC9629059

[B146] Sanjuan M. A. Dillon C. P. Tait S. W. G. Moshiach S. Dorsey F. Connell S. (2007). Toll-like receptor signalling in macrophages links the autophagy pathway to phagocytosis. *Nature* 450 1253–1257. 10.1038/nature06421 18097414

[B147] Sawa-Makarska J. Baumann V. Coudevylle N. Von B. Nogellova V. Abert C. (2020). Reconstitution of autophagosome nucleation defines Atg9 vesicles as seeds for membrane formation. *Science* 369:eaaz7714. 10.1126/science.aaz7714 32883836 PMC7610778

[B148] Scherz-Shouval R. Elazar Z. (2011). Regulation of autophagy by Ros: Physiology and pathology. *Trends Biochem. Sci.* 36 30–38. 10.1016/j.tibs.2010.07.007 20728362

[B149] Schiess M. C. Barnes J. L. Ellmore T. M. Poindexter B. J. Dinh K. Bick R. J. (2010). Csf from Parkinson disease patients differentially affects cultured microglia and astrocytes. *BMC Neurosci.* 11:151. 10.1186/1471-2202-11-151 21114836 PMC3012671

[B150] Schwarcz R. Guidetti P. Sathyasaikumar K. V. Muchowski P. J. (2010). Of mice, rats and men: Revisiting the quinolinic acid hypothesis of Huntington’s disease. *Prog. Neurobiol.* 90 230–245. 10.1016/j.pneurobio.2009.04.005 19394403 PMC2829333

[B151] Sebastiani G. Almeida-Toledano L. Serra-Delgado M. Navarro-Tapia E. Sailer S. Valverde O. (2021). Therapeutic effects of catechins in less common neurological and neurodegenerative disorders. *Nutrients* 13:33. 10.3390/nu13072232 34209677 PMC8308206

[B152] Sestito S. Daniele S. Pietrobono D. Citi V. Bellusci L. Chiellini G. (2019). Memantine prodrug as a new agent for Alzheimer’s disease. *Sci. Rep.* 9:11. 10.1038/s41598-019-40925-8 30874573 PMC6420495

[B153] Shu X. D. Sun Y. M. Sun X. Y. Zhou Y. Z. Bian Y. Q. Shu Z. M. (2019). The effect of fluoxetine on astrocyte autophagy flux and injured mitochondria clearance in a mouse model of depression. *Cell Death Dis.* 10:16. 10.1038/s41419-019-1813-9 31371719 PMC6675792

[B154] Sierra A. Abiega O. Shahraz A. Neumann H. (2013). Janus-faced microglia: Beneficial and detrimental consequences of microglial phagocytosis. *Front. Cell. Neurosci.* 7:22. 10.3389/fncel.2013.00006 23386811 PMC3558702

[B155] Singh D. P. Herrera C. E. Singh B. Singh S. Singh R. K. Kumar R. (2018). Graphene oxide: An efficient material and recent approach for biotechnological and biomedical applications. *Mater. Sci. Eng. C Mater. Biol. Appl.* 86 173–197. 10.1016/j.msec.2018.01.004 29525091

[B156] Sliter D. A. Martinez J. Hao L. Chen X. Sun N. Fischer T. D. (2018). Parkin and Pink1 mitigate Sting-induced inflammation. *Nature* 561 258–262. 10.1038/s41586-018-0448-9 30135585 PMC7362342

[B157] Su P. Zhang J. Wang D. Zhao F. Cao Z. Aschner M. (2016). The role of autophagy in modulation of neuroinflammation in microglia. *Neuroscience* 319 155–167. 10.1016/j.neuroscience.2016.01.035 26827945

[B158] Sun Q. M. Zhang J. Fan W. L. Wong K. N. Ding X. J. Chen S. (2011). The run domain of rubicon is C binding. Lipid kinase inhibition, and autophagy suppression. *J. Biol. Chem.* 286 185–191. 10.1074/jbc.M110.126425 21062745 PMC3012973

[B159] Tabet N. (2006). Acetylcholinesterase inhibitors for Alzheimer’s disease: Anti-inflammatories in acetylcholine clothing. *Age Ageing* 35 336–338. 10.1093/ageing/afl027 16788077

[B160] Tai Y. F. Pavese N. Gerhard A. Tabrizi S. J. Barker R. A. Brooks D. J. (2007). Microglial activation in presymptomatic Huntington’s disease gene carriers. *Brain* 130 1759–1766. 10.1093/brain/awm044 17400599

[B161] Talbot P. R. Goulding P. J. Lloyd J. J. Snowden J. S. Neary D. Testa H. J. (1995). Inter-relation between “classic” motor neuron disease and frontotemporal dementia: Neuropsychological and single photon emission computed tomography study. *J. Neurol. Neurosurg. Psychiatry* 58 541–547. 10.1136/jnnp.58.5.541 7745399 PMC1073482

[B162] Tang Y. Le W. D. (2016). Differential roles of M1 and M2 microglia in neurodegenerative diseases. *Mol. Neurobiol.* 53 1181–1194. 10.1007/s12035-014-9070-5 25598354

[B163] Tasset I. Cuervo A. M. (2016). Role of chaperone-mediated autophagy in metabolism. *FEBS J.* 283 2403–2413. 10.1111/febs.13677 26854402 PMC4935551

[B164] Torre D. Speranza F. Giola M. Matteelli A. Tambini R. Biondi G. (2002). Role of Th1 and Th2 cytokines in immune response to uncomplicated Plasmodium falciparum malaria. *Clin. Diag. Lab. Immunol.* 9 348–351. 10.1128/CDLI.9.2.348-351.2002 11874876 PMC119927

[B165] Toulouse A. Sullivan A. M. (2008). Progress in Parkinson’s disease - where do we stand? *Prog. Neurobiol.* 85 376–392. 10.1016/j.pneurobio.2008.05.003 18582530

[B166] Tremblay M. E. Stevens B. Sierra A. Wake H. Bessis A. Nimmerjahn A. (2011). The role of microglia in the healthy brain. *J. Neurosci.* 31 16064–16069. 10.1523/JNEUROSCI.4158-11.2011 22072657 PMC6633221

[B167] Turco E. Witt M. Abert C. Bock-Bierbaum T. Su M. Y. Trapannone R. (2019). Fip200 claw domain binding to p62 promotes autophagosome formation at ubiquitin condensates. *Mol. Cell* 74 330–346. 10.1016/j.molcel.2019.01.035 30853400 PMC6477179

[B168] Ullah F. Liang A. Rangel A. Gyengesi E. Niedermayer G. Munch G. (2017). High bioavailability curcumin: An anti-inflammatory and neurosupportive bioactive nutrient for neurodegenerative diseases characterized by chronic neuroinflammation. *Arch. Toxicol.* 91 1623–1634. 10.1007/s00204-017-1939-4 28204864

[B169] Vamecq J. Latruffe N. (1999). Medical significance of peroxisome proliferator-activated receptors. *Lancet (London, England)* 354 141–148. 10.1016/S0140-6736(98)10364-1 10408502

[B170] Vargas J. N. S. Wang C. X. Bunker E. Hao L. Maric D. Schiavo G. (2019). Spatiotemporal control of Ulk1 activation by Ndp52 and Tbk1 during selective autophagy. *Mol. Cell* 74 347–362. 10.1016/j.molcel.2019.02.010 30853401 PMC6642318

[B171] Walczak M. Martens S. (2013). Dissecting the role of the Atg12-Atg5-Atg16 complex during autophagosome formation. *Autophagy* 9 424–425. 10.4161/auto.22931 23321721 PMC3590266

[B172] Walensky L. D. (2006). Bcl-2 in the crosshairs: Tipping the balance of life and death. *Cell Death Differ.* 13 1339–1350. 10.1038/sj.cdd.4401992 16763614

[B173] Walker F. O. (2007). Huntington’s disease. *Lancet* 369 218–228. 10.1016/S0140-6736(07)60111-1 17240289

[B174] Wang Y. M. Cella M. Mallinson K. Ulrich J. D. Young K. L. Robinette M. L. (2015). Trem2 lipid sensing sustains the microglial response in an Alzheimer’s disease model. *Cell* 160 1061–1071. 10.1016/j.cell.2015.01.049 25728668 PMC4477963

[B175] Watkins P. B. Zimmerman H. J. Knapp M. J. Gracon S. I. Lewis K. W. (1994). Hepatotoxic effects of tacrine administration in patients with Alzheimer’s disease. *Jama* 271 992–998. 10.1001/jama.1994.03510370044030 8139084

[B176] Wu A. G. Zhou X. G. Qiao G. Yu L. Tang Y. Yan L. (2021). Targeting microglial autophagic degradation in Nlrp3 inflammasome-mediated neurodegenerative diseases. *Ageing Res. Rev.* 65:21. 10.1016/j.arr.2020.101202 33161129

[B177] Wu T. T. Li W. M. Yao Y. M. (2016). Interactions between autophagy and inhibitory cytokines. *Int. J. Biol. Sci.* 12 884–897. 10.7150/ijbs.15194 27313501 PMC4910606

[B178] Xilouri M. Stefanis L. (2016). Chaperone mediated autophagy in aging: Starve to prosper. *Ageing Res. Rev.* 32 13–21. 10.1016/j.arr.2016.07.001 27484893

[B179] Xu J. Z. Wang Y. F. Tan X. R. Jing H. J. (2012). Micrornas in autophagy and their emerging roles in crosstalk with apoptosis. *Autophagy* 8 873–882. 10.4161/auto.19629 22441107 PMC3427253

[B180] Xu Y. D. Cui C. Sun M. F. Zhu Y. L. Chu M. Shi Y. W. (2017). Neuroprotective effects of loganin on Mptp-Induced Parkinson’s disease mice: Neurochemistry. Glial reaction and autophagy studies. *J. Cell. Biochem.* 118 3495–3510. 10.1002/jcb.26010 28338241

[B181] Yamamoto A. Cremona M. L. Rothman J. E. (2006). Autophagy-mediated clearance of huntingtin aggregates triggered by the insulin-signaling pathway. *J. Cell Biol.* 172 719–731. 10.1083/jcb.200510065 16505167 PMC2063704

[B182] Yan Q. T. Han C. J. Wang G. H. Waddington J. L. Zheng L. T. Zhen X. C. (2017). Activation of Ampk/mtorc1-mediated autophagy by metformin reverses Clk1 deficiency-sensitized dopaminergic neuronal death. *Mol. Pharmacol.* 92 640–652. 10.1124/mol.117.109512 29025968

[B183] Yang D. S. Stavrides P. Mohan P. S. Kaushik S. Kumar A. Ohno M. (2011). Reversal of autophagy dysfunction in the Tgcrnd8 mouse model of Alzheimer’s disease ameliorates amyloid pathologies and memory deficits. *Brain* 134 258–277. 10.1093/brain/awq341 21186265 PMC3009842

[B184] Yang Z. F. Klionsky D. J. (2010). Mammalian autophagy: Core molecular machinery and signaling regulation. *Curr. Opin. Cell Biol.* 22 124–131. 20034776 10.1016/j.ceb.2009.11.014 PMC2854249

[B185] Yin P. Wang X. Wang S. Wei Y. F. Feng J. C. Zhu M. Q. (2019). Maresin 1 improves cognitive decline and ameliorates inflammation in a mouse model of Alzheimer’s disease. *Front. Cell. Neurosci.* 13:466. 10.3389/fncel.2019.00466 31680874 PMC6803487

[B186] Yu J. W. Lee M. S. (2016). Mitochondria and the Nlrp3 inflammasome: Physiological and pathological relevance. *Arch. Pharm. Res.* 39 1503–1518. 10.1007/s12272-016-0827-4 27600432

[B187] Yuan J. X. Liu H. H. Zhang H. Wang T. T. Zheng Q. Li Z. (2022). Controlled activation of Trpv1 channels on microglia to boost their autophagy for clearance of alpha-synuclein and enhance therapy of Parkinson’s disease. *Adv. Mater.* 34:13. 10.1002/adma.202108435 35023596

[B188] Yuan Y. J. Chen Y. N. Peng T. Q. Li L. Zhu W. Z. Liu F. (2019). Mitochondrial Ros-induced lysosomal dysfunction impairs autophagic flux and contributes to M1 macrophage polarization in a diabetic condition. *Clin. Sci.* 133 1759–1777. 10.1042/CS20190672 31383716

[B189] Zaffagnini G. Martens S. (2016). Mechanisms of selective autophagy. *J. Mol. Biol.* 428 1714–1724. 10.1016/j.jmb.2016.02.004 26876603 PMC4871809

[B190] Zeng Q. Siu W. S. Li L. M. Jin Y. Liang S. Y. Cao M. Q. (2019). Autophagy in Alzheimer’s disease and promising modulatory effects of herbal medicine. *Exp. Gerontol.* 119 100–110. 10.1016/j.exger.2019.01.027 30710681

[B191] Zhang Q. Zhou J. Shen M. Xu H. Yu S. Cheng Q. (2020). Pyrroloquinoline quinone inhibits rotenone-induced microglia inflammation by enhancing autophagy. *Molecules* 25:15. 10.3390/molecules25194359 32977419 PMC7582530

[B192] Zhong G. Long H. Zhou T. Liu Y. Zhao J. Han J. (2022). Blood-brain barrier Permeable nanoparticles for Alzheimer’s disease treatment by selective mitophagy of microglia. *Biomaterials* 288 121690. 10.1016/j.biomaterials.2022.121690 35965114

[B193] Zubova S. G. Suvorova I. I. Karpenko M. N. (2022). Macrophage and microglia polarization: Focus on autophagy-dependent reprogramming. *Front. Biosci.* 14:3. 10.31083/j.fbs1401003 35320914

